# Ruthenium Olefin Metathesis Catalysts Featuring N-Heterocyclic Carbene Ligands Tagged with Isonicotinic and 4-(Dimethylamino)benzoic Acid Rests: Evaluation of a Modular Synthetic Strategy

**DOI:** 10.3390/molecules26175220

**Published:** 2021-08-28

**Authors:** Stefan Czarnocki, Louis Monsigny, Michał Sienkiewicz, Anna Kajetanowicz, Karol Grela

**Affiliations:** Biological and Chemical Research Centre, Faculty of Chemistry, University of Warsaw, Żwirki i Wigury Street 101, 02-089 Warsaw, Poland; stefanczarnocki@gmail.com (S.C.); l.monsigny@cent.uw.edu.pl (L.M.); mikes@uwb.edu.pl (M.S.)

**Keywords:** olefin metathesis, ruthenium, N-heterocyclic carbenes, catalysis

## Abstract

A modular and flexible strategy towards the synthesis of N-heterocyclic carbene (NHC) ligands bearing Brønsted base tags has been proposed and then adopted in the preparation of two tagged NHC ligands bearing rests of isonicotinic and 4-(dimethylamino)benzoic acids. Such tagged NHC ligands represent an attractive starting point for the synthesis of olefin metathesis ruthenium catalysts tagged in non-dissociating ligands. The influence of the Brønsted basic tags on the activity of such obtained olefin metathesis catalysts has been studied.

## 1. Introduction

**Figure 1:**
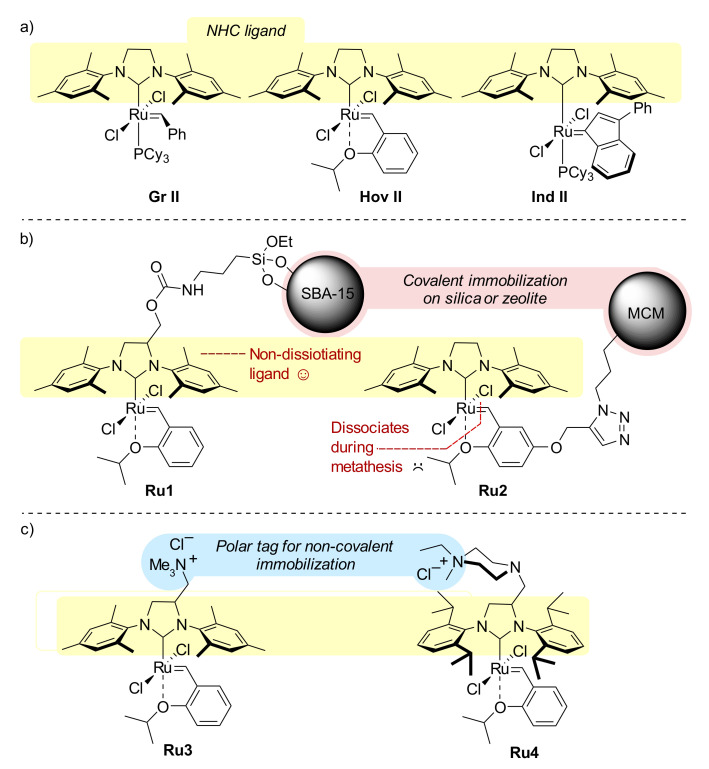
Selected ruthenium olefin metathesis catalysts: (**a**) Grubbs, Hoveyda-Grubbs, and indenylidene type general-use complexes. (**b**) Examples of complexes covalently bound to the support. (**c**) Examples of commercially available complexes bearing polar quaternary ammonium tags for non-covalent attachment to the support. Cy = cyclohexyl, SBA-15 = Santa Barbara Amorphous-15, a highly stable mesoporous silica sieve, MCM = class of micro- and mesoporous zeolites.

Olefin metathesis has been well established as a key route in the formation of C-C double bonds in the canon of modern organometallic chemistry [1,2]. Its privileged role would not be possible without the development of well-defined ruthenium complexes, especially stable and easily adjustable Grubbs, Hoveyda–Grubbs, and indenylidene type second-generation catalysts possessing N-heterocyclic carbenes (NHC) (Figure 1, top) [3,4]. Besides improving the control over the (*E*)/(*Z*) selectivity [5,6] and substrate scope of olefin metathesis reactions themselves [7,8,9,10,11], considerable process-related efforts have been undertaken to increase their attractiveness for industrial applications with respect to catalyst activity and stability, possibility of its recycling, and minimizing the level of toxic and polluting metal contaminants in the final products [12,13,14]. An attractive solution for the latter issue is the heterogenization of the catalyst on solid (or liquid) supports. Over the past decades, both covalent [15,16] and non-covalent [17,18] anchoring of the catalyst to the support has been explored (Figure 1) [19,20,21]. Covalent immobilization is often accompanied with the costly development of sophisticated linkers, in catalysts as well as supports [22]. Once spent, the catalyst cannot be easily recharged/replaced on the support, further increasing costs [12,22]. Moreover, most of the covalently linked catalysts reported show diminished activity, and their reusability remains moderate. Non-covalent binding can rely on electrostatic interactions [23,24,25,26,27], reversible π-π interactions [28,29], or physio-sorption on porous materials, such as silica [30,31,32,33,34,35], charcoal, cellulose, wool, and filter paper [36], dendrimers [37,38,39], SBA-15 [40], or MOF [41,42,43].

Despite the preparation of non-covalently immobilized catalysts is in general considered to be simpler and more straightforward compared to the manufacturing of their covalently bound analogues [21], the preparation of tagged catalysts is in any case complicated from the synthetic point of view. For example, the synthesis of quaternary ammonium tagged catalysts, such as **Ru3** or **Ru4**, consists of 3–4 additional synthetic steps compared to the synthesis of their most close non-tagged analogues [31,44,45] and is sometimes associated with problems [46,47]. 

Therefore, we believe that there is a space for developing a more universal and direct method for the synthesis of ligands bearing a handle for various tags. Here, we present a modular and flexible strategy towards N-heterocyclic carbene (NHC) ligands bearing Brønsted base tags that is based on ester formation (benzoylation) [48]. Such tagged NHC ligands bearing rests of isonicotinic and 4-(dimethylamino)benzoic acids were used in synthesis of the corresponding olefin metathesis ruthenium catalysts. The non-ionic nature of the tagged fragments shall help to circumvent some previously noted problems during the synthesis of quaternary ammonium precursors (vide supra), while their basicity shall open a venue for the non-covalent immobilization of such obtained catalysts (not studied in this work).

## 2. Results

It is well known that the introduction of a group (tag) that could non-covalently bind to various solid supports would allow for the reversible immobilization of the corresponding catalyst opening the possibility of its heterogeneous use. At the same time, the introduction of such a tag could allow the catalyst easier removal after reaction, if the system is used under homogeneous conditions [21]. The most popular method for tagging of ruthenium benzylidene catalysts is to modify the NHC ligand or the benzylidene fragment [17,18,21]. As the benzylidene ligand dissociates in the first step of the catalytic cycle, the NHC ligand—the one which is bound to the ruthenium throughout the whole process—was chosen by us as the preferred handle for a tag. 

**Figure 2:**
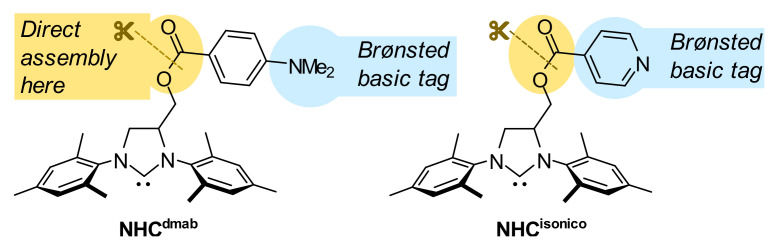
Planned new tagged NHC ligands bearing basic Brønsted sites.

Our present goal was to blueprint and then execute a direct, modular synthetic protocol for manufacturing tagged ruthenium catalysts. In our plan, the basic nitrogen-containing tags could be protonated in the presence of Brønsted acids, a feature that we plan to utilize later for the non-covalent immobilization of the ruthenium complexes (not studied in this report). Looking for the most convenient synthetic route leading to such tagged NHC ligands, we envisioned that the ester formation reaction shall be used, as it would allow to install various carboxylic acid rests (modularity), following a relatively simple and standard synthetic protocol. The structures of two imagined tagged NHC ligands (referred as NHC^dmab^ and NHC^isonico^) with Brønsted basic tags are shown in Figure 2. They contain rests of isonicotinic and 4-(dimethylamino)benzoic acids attached to a CH_2_OH handle placed in position 4 of the imidazolidin-2-ylidene ligand backbone. 

**Scheme 1:**
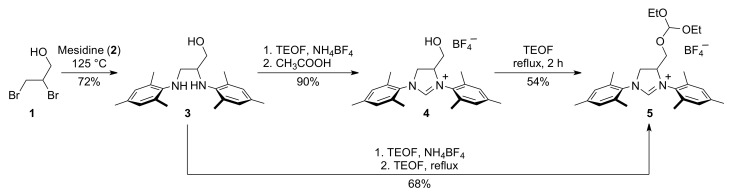
Preparation of NHC ligand precursors **4** and **5**. TEOF = triethyl orthoformate, mesidine = 2,4,6-trimethylaniline.

To reduce this plan to reality, salt **4** was prepared in three-step procedure (Scheme 1). First, 2,3-dibromopropanol (**1**) was treated with 2,4,5-trimethylaniline (mesidine, **2**) to yield diamine **3** which was treated with ammonium tetrafluoroborate in refluxing triethyl orthoformate (TEOF) to give the desired product **4** in 90% yield. Next, the hydroxyl group was protected, to assure compatibility with the next steps that consist of deprotonation of the NHC precursor with a strong base and then metallation. In related syntheses described in the literature, usually silicon protecting groups or tetrahydropyran (THP) are used for this purpose [49,50,51]. In our synthesis, however, we simply used triethyl orthoformate as a cheap “temporal” protecting group, providing salt **5** in 54% yield.

Further improvement of the synthesis of **5** led us to consider the direct formation of the protected ligand from the precursor **3**. To do so, the treatment of **3** with NH_4_BF_4_ in refluxing TEOF was undertaken to form first a mixture of imidazolium salts **4** + **5** which was transformed one-pot to **5**. After the evaporation of the volatiles, the crude mixture was composed of product **4** and corresponding protected ligand **5** (ratio **4**/**5**: 7/3). Instead of working up the crude mixture with an acid to obtain **4**, we forced the protection of **4** in situ by adding a fresh portion of TEOF and heat the resulting mixture at 120 °C for 2 h. Such procedure led to the formation of the protected imidazolium salt which has been isolated in 68% yield. Of interest, the iteration of the second step on the crude mixture did not improve further the yield of this reaction and led to corresponding product in similar yield than previously after purification (68%). It shall be noted that this one-pot procedure is not only more convenient experimentally, but also provides higher yield (68%) as compared with the two-step procedure, which gave **5** in 49% total yield.

**Scheme 2:**
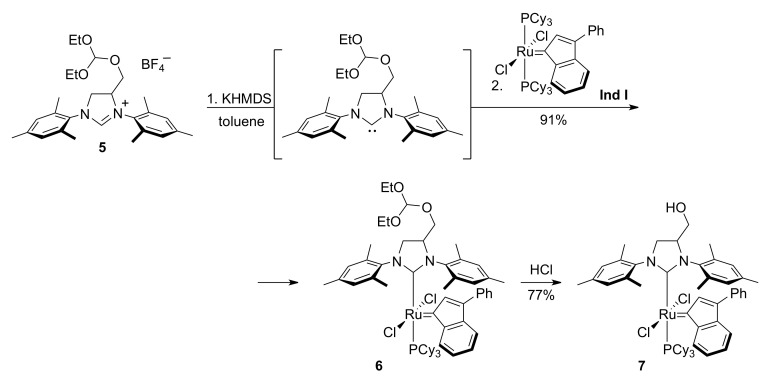
Preparation of ruthenium complex **7** possessing free OH group.

With the ligand precursor **5** in hand, we proceeded to synthesize complex **7** (Scheme 2). To do so, the free carbene was generated in situ by treatment of **5** with KHMDS (potassium bis(trimethylsilyl)amide) and the resulting species reacted with **Ind I** to provide a protected catalyst **6** with a very good yield of 91% (Scheme 2). Two drops of HCl in dioxane (4M solution) were used to obtain “unprotected” complex **7** possessing free OH group with good yield. 

**Scheme 3:**
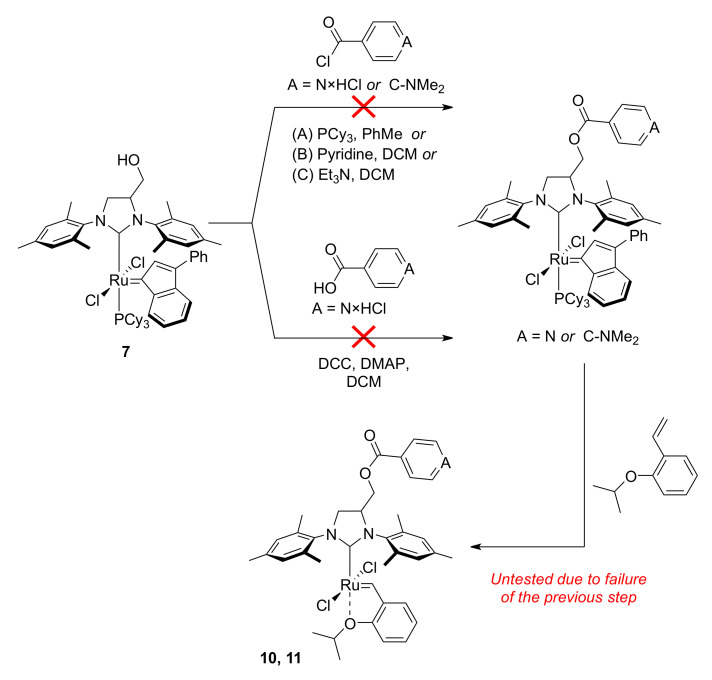
Failed direct esterification of **7**.

In the next step, we tried direct esterification of ruthenium complex **7** with 4-(dimethylamino)benzoyl chloride to form the corresponding indenylidene type complex (Scheme 3). The obtained complex would be not only a potentially immobilizable catalyst, but we planned to use it as a precursor for various functionalized Hoveyda–Grubbs-type catalysts [9]. To do esterification of ruthenium complex **7**, we adopted the conditions developed previously by Buchmeiser and Fürstner for acylation of related HO-tagged Ru complex with AcCl, that utilizes tricyclohexylphosphine as a mild base to neutralize the produced HCl [52]. Unfortunately, despite numerous efforts, the direct benzoylation of **7** with 4-(dimethylamino)benzoyl chloride was unsuccessful in our hands and the desired product was not formed. Next, a number of attempts for the esterification of complex **7** with both 4-(dimethylamino)benzoyl chloride and isonicotinoyl chloride hydrochloride were undertaken by us using different bases such as pyridine and triethylamine. While the use of pyridine was found to decompose rapidly complex **7**, the reaction in the presence of triethylamine led to the formation of the expected products which has been observed both on TLC and in ^1^H–NMR spectra of the crude reaction mixture. Unfortunately, almost as they were formed, the products rapidly decomposed during the workup process involving column chromatography. Finally, we also tried the classical coupling of complex **7** and the isonicotinic acid in presence of dicyclohexylcarbodiimide (DCC) and a catalytic quantity of 4-dimethylaminopyridine (DMAP). Here again, the desired product was not formed in such conditions and only the decomposition of complex **7** was observed (Scheme 3).

**Scheme 4:**
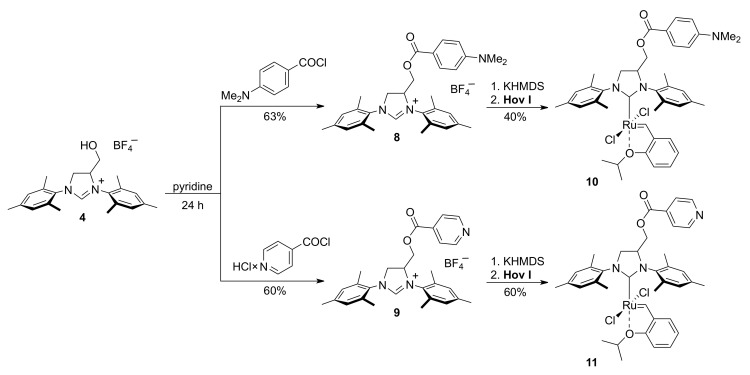
Preparation of NHC ligand precursors **8** and **9** and Hoveyda-Grubbs type complexes **10** and **11**.

At this point, we took a step back and decided to first obtain an NHC ligand precursor containing already the tag fragments and then metallated it with Ru precursor to obtain the corresponding ruthenium complexes. To do so, we performed an esterification reaction between salt **4** and two acid chlorides, (4-(dimethylamino)benzoyl chloride and isonicotinoyl chloride hydrochloride). The process was performed in pyridine at room temperature for 16 h, giving salts **8** and **9** in 63 and 60% yield, respectively. The next step was to form the corresponding NHC-Ru alkylidene complexes **10** and **11**, which were synthesized in 40% and 60% yield, respectively (after silica gel chromatography). We are satisfied to see that their synthesis was straightforward and followed the standard procedure consisting of deprotonation of the NHC ligand precursor with KHMDS and treatment of the resulting free carbene with the Hoveyda first generation complex (Scheme 4).

**Scheme 5:**
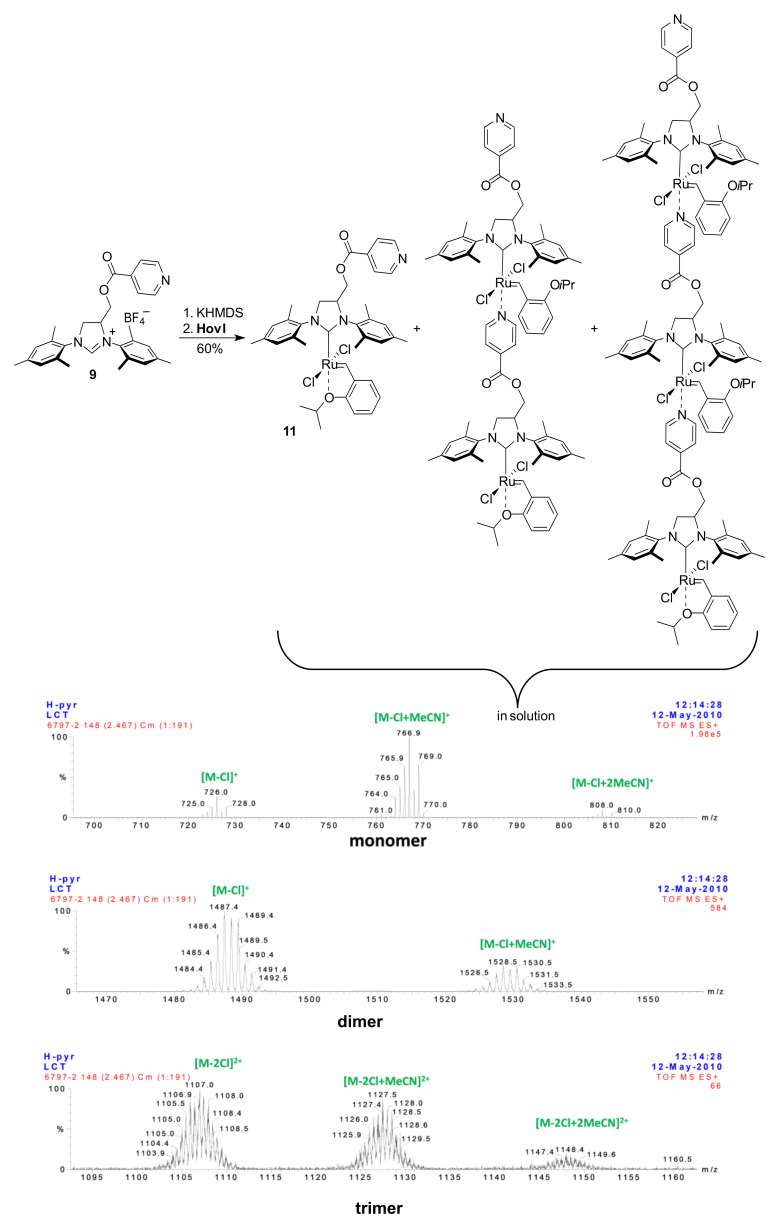
Formation of dimers and trimers of complex **11** and their MS ESI spectra.

The stability of such obtained complexes, which were isolated as green (**10**) and orange-brownish (**11**) microcrystalline solids was very high, which is typical for Hoveyda–Grubbs complexes. We noted that they are stable in non-degassed deuterated DCM (after two days no sign of decomposition was observed) and can be stored for several months in a solid form (+4 °C, under argon) without any sign of decomposition. In ^1^H-NMR spectrum, the benzylidene proton signals of **10** and **11** were observed at 16.51 and 16.47 ppm, respectively, which is also a typical value for Hoveyda–Grubbs complexes. However, an atypical feature was observed in the ESI MS spectra of complex **11**, suggesting that this catalyst exists in solution in the form of dimers, trimers, and in part as higher oligomers, probably formed via pyridine tag → Ru chelation (Scheme 5).

**Figure 3:**
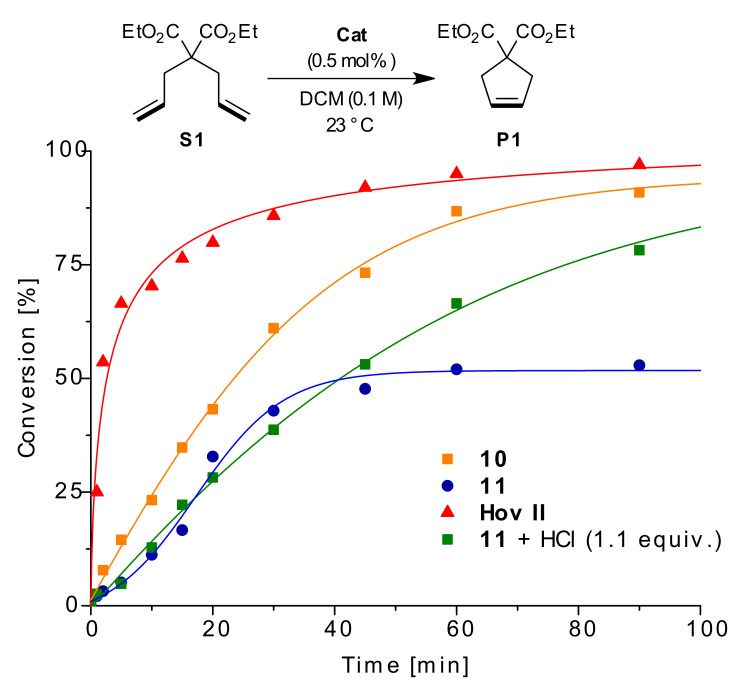
Conversion to product **P1** using catalysts **10**, **11**, and **Hov II**. Conversions according to ^1^H-NMR, curves are visual aid only.

Next, we were interested to see how the presence of Brønsted base fragments influenced the catalytic activity of the formed complexes. To do so, we used a model ring-closing metathesis (RCM) reaction of diethyl diallylmalonate (**S1**) leading to the formation of the cyclic product **P1 [53]**. We set out to compare the newly prepared complexes **10** and **11** with the parent Hoveyda–Grubbs catalyst (**Hov II**) which has found broad application in metathesis and thus is a commonly accepted benchmark [39]. To limit the reaction rate to a level useful for comparison, relatively mild conditions (0.5 mol% of catalyst, 23 °C) were employed. The course of the reaction was monitored by ^1^H-NMR. Interestingly, we found a difference between the behavior of catalysts **10** and **11**. The first one, containing a dimethylaniline fragment in the tag, proved to be more reactive than the pyridine tagged complex **11**. Strongly limited activity of the latter can be explained by self-poisoning of the Lewis basic pyridine fragment, which by coordinating to the Ru center can arrest the catalyst blocking it from entering into the catalytic cycle. As illustrated in Figure 3, there is a dramatic improvement in the activity of catalyst **11** in the presence of mineral acid (1.1 equivalent HCl in dioxane), probably due to protonation of the basic pyridine center and inhibition of catalyst decomposition.

**Scheme 6:**
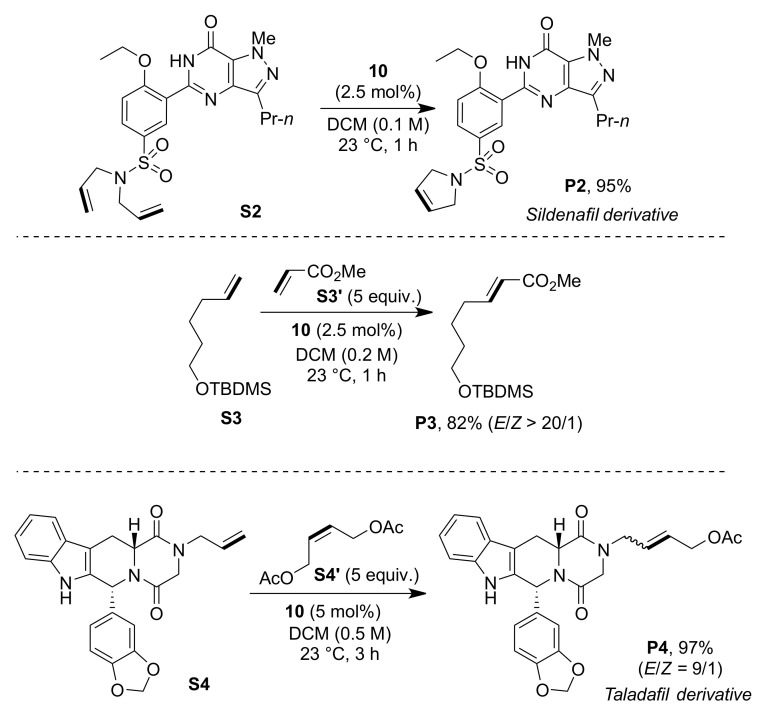
Metathesis reactions of APIs **S2**, **S3**, and **S4** catalyzed by **10** (isolated yields of pure substances).

Having in hand catalyst **10** demonstrating a good reactivity in RCM of **S1** without any additives, we decided to explore the scope of such complex in challenging RCM reaction of highly functionalized substrates such as Sildenafil derivative **S2** (Scheme 6). Because of the density of functionalities, such RCM has been undertaken with 2.5 mol% of **10** in DCM (0.1 M). Gratifyingly, the RCM rapidly occurred in these conditions, and complex **10** promoted the RCM of **S2** to afford to the corresponding product **P2** with an excellent yield of 95%. Further investigations of the scope led us to consider reaction of cross metathesis (CM) of the silylated hex-5-en-1-ol derivative (**S3**) with a challenging electron poor CM partner, methyl acrylate (**S3′**). The reaction reached completion with 2.5 mol% of the catalyst **10** in DCM (0.2 M) within 1 h to yield selectively to the corresponding product **P3** (82%) for which only the *E* isomer has been observed. Based on this result, CM-reluctant Taladafil derivative **P4** has been considered a substrate for the CM reaction promoted by **10**. We reported recently such CM reaction of **P4** with (*Z*)-1,4-diacetoxybutene **S4′** (5 equivalents) as cross partner with 1 mol% of a sulfonamide activated Hoveyda type Ru-based catalyst [54]. In such conditions, the CM reaction at room temperature afford the corresponding product in 68% with an *E*/*Z* ratio of 95/5 within 15 h. Based on this report, the RCM of **S4** (1 equivalent, 0.5 M in DCM) was undertaken with 5 equivalents of the cross partner **S4′** and with 5 mol% of complex **10**. Remarkably, the CM reaction took place at room temperature and reached completion within 3 h in these conditions. A simple purification through column chromatography on silica led to the isolation of the expected product in 97% yield and an *E*/*Z* ratio of 9/1. Of high interest, complex **10** surpassed in term of productivity the sulfonamide activated Hoveyda–Grubbs catalysts at the expense of the *E*/*Z* selectivity.

## 3. Experimental Data

All reagents were purchased from Sigma-Aldrich Chemical Company and used without further purification. Toluene was distilled over potassium under an argon atmosphere. Analytical thin-layer chromatography (TLC) was performed using silica gel 60 F_254_ pre-coated plates (0.25 mm thickness) with a fluorescent indicator. Visualization of TLC plates was performed using UV light and either KMnO_4_ or I_2_ stains. Flash chromatography was performed using silica gel 60 (230–400 mesh). NMR spectra were recorded in CDCl_3_ or C_6_D_6_ on a Varian VNMRS 500 MHz spectrometer. The ^1^H and ^13^C chemical shifts are referenced to SiMe_4_ (δ = 0 ppm). The following abbreviations are used in reporting NMR data: s (singlet), d (doublet), t (triplet), q (quartet), m (multiplet), br (broad). Coupling constants (*J*) are given in Hz. Spectra are reported as follows: chemical shift (δ, ppm), multiplicity, integration, coupling constants (Hz). IR spectra were recorded on a Perkin-Elmer Spectrum One FTIR spectrometer with diamond ATR accessory. Wave numbers are in cm^−1^. Micro-analyses were made using Vario EL III apparatus. MS spectra were recorded by LCT Micromass. Experimental details for the unsuccessful direct esterification of complex **7**, the metathesis reactions and spectroscopic data can be found in the Supplementary Materials.

### 3.1. N,N′-Dimesityl-2,3-diamino-1-propanol (***3***) 

2,3-Dibromopropanol (61.9 g, 284 mmol) was treated with 2,4,6-trimethylaniline (160.0 mL, 1136 mmol). After stirring at 125 °C for 24 h, DCM and an aqueous 15% sodium hydroxide solution (34.0 g, 852 mmol) were added and the reaction mixture was stirred until all solid was completely dissolved. The organic layer was separated, washed with water, dried over sodium sulphate, and concentrated. Crystallization from hexane gave the product as a colorless solid (66.8 g, 72%). ^1^H-NMR (500 MHz, CDCl_3_) δ 6.81 (d, *J* = 9.8, Hz, 4H), 3.94 (dd, *J* = 11.0, 2.7 Hz, 1H), 3.82 (ddd, *J* = 11.0, 4.2, 1.2 Hz, 1H), 3.54 (br s, 3H), 3.41–3.35 (m, 1H), 3.21 (ddd, *J* = 12.0, 5.1, 1.2 Hz, 1H), 2.98 (dd, *J* = 12.0, 3.9 Hz, 1H), 2.28 (s, 6H), 2.22 (s, 3H), 2.21 (s, 3H), 2.16 (s, 6H).

### 3.2. 1,3-Bis(1-mesityl)-4,5-dihydro-4-hydroxymethyl-1H-imidazol-3-ium Tetrafluoroborate (***4***)

*N,N′*-Dimesityl-2,3-diamino-1-propanol [55] (52.5 g, 161 mmol) and ammonium tetrafluoroborate (16.9 g, 161 mmol) were dissolved in triethyl orthoformate (66.9 mL, 402 mmol). After stirring at 120 °C for 2 h, the reaction mixture was cooled and the volatiles were removed under vacuum. The resulting yellow oil was treated with acetic acid. Crystallization from DCM/CCl_4_ gave the product as a colorless solid (61.5 g, 90%). ^1^H-NMR (500 MHz, CDCl_3_) δ 7.76 (s, 1H), 6.97–6.90 (m, 4H), 4.93–4.86 (m, 1H), 4.49 (s, 1H), 4.47 (d, *J* = 2.2 Hz, 1H), 3.82–3.75 (m, 1H), 3.60 (d, *J* = 11.2 Hz, 2H), 2.40 (s, 3H), 2.33 (s, 3H), 2.32–2.28 (m, 6H), 2.27 (s, 6H).

### 3.3. 1,3-Bis(1-mesityl)-4,5-dihydro-4-diethyloxymethoxymethyl-1H-imidazol-3-ium Tetrafluoroborate (***5***)

1,3-Bis(1-mesityl)-4,5-dihydro-4-hydroxymethyl-1*H*-imidazol-3-ium tetrafluoroborate [55] (30.0 g, 70.7 mmol) was dissolved in triethyl orthoformate (35.3 mL, 212 mmol). After stirring at 100 °C for 2 h, the reaction mixture was cooled and the volatiles were removed under vacuum. Purification by flash column chromatography (10% DCM/acetone) gave a white solid (20.2 g, 54%). ^1^H-NMR (500 MHz, CDCl_3_) δ 7.97 (s, 1H), 6.99–6.75 (m, 4H), 5.17 (s, 1H), 5.17–5.12 (m, 1H), 4.65 (t, *J* = 12.2 Hz, 1H), 4.37 (dd, *J* = 12.2, 8.1 Hz, 1H), 3.79 (dd, *J* = 12.0, 2.9 Hz, 1H), 3.64–3.45 (m, 5H), 2.40 (s, 3H), 2.36 (s, 6H), 2.32 (brs, 3H), 2.29 (s, 6H), 1.20–1.12 (m, 6H). ^13^C NMR (125 MHz, CDCl_3_) δ 158.8, 140.8, 140.5, 136.3, 135.6, 130.7, 130.5, 130.4, 128.6, 113.2, 63.8, 61.1, 60.8, 60.7, 52.8, 21.3, 21.2, 18.4, 18.0, 17.7, 15.1. HRMS (ESI): M^+^_,_ found 439.2972, requires C_27_H_39_N_2_O_3_ 439.2961.

### 3.4. Alternative Synthesis of 1,3-bis(1-mesityl)-4,5-dihydro-4-diethyloxymethoxymethyl-1H-imidazol-3-ium Tetrafluoroborate (***5***)

*N,N′*-Dimesityl-2,3-diamino-1-propanol (1.0 g, 3.1 mmol) and ammonium tetrafluoroborate (0.321 g, 3.1 mmol, 1 equivalent) were dissolved in triethyl orthoformate (1.3 mL, 1.16 mg, 7.66 mmol, 2.5 equivalents). After stirring at 120 °C for 2 h, the reaction mixture was cooled and the volatiles were removed under vacuum. The resulting brown solid was dissolved in triethyl orthoformate (2.6 mL, 2.32 mg, 15.32 mmol, 5 equivalent). After stirring at 120 °C for 2 h, the reaction mixture was cooled and the volatiles were removed under vacuum. Purification by flash column chromatography (10% DCM/acetone) gave a white solid (735 mg, 68%).

### 3.5. Catalyst ***6***

To a solution of ligand **5** (1.053 g, 2.00 mmol) in toluene (10 mL) was added a solution of KHMDS in THF (8.0 mL, 2.00 mmol). After stirring at rt for 20 min, **Ind I** (0.925 g, 1.00 mmol) was added and stirring was continued for 20 min at 70 °C. The reaction mixture was then concentrated in vacuo. Purification by flash column chromatography (5% ethyl acetate/cyclohexane) gave a brown solid (1.650 g, 91%). ^1^H-NMR (500 MHz, Benzene-*d_6_*) δ 9.24–9.04 (m, 1H), 7.94–7.80 (m, 1H), 7.38–7.17 (m, 5H), 7.15–6.93 (m, 5H), 6.52–6.41 (m, 1H), 6.06–5.96 (m, 1H), 4.92–4.76 (m, 1H), 4.34–4.12 (m, 1H) 3.94–3.48 (m, 3H), 3.44–3.20 (m, 4H), 3.08–2.76 (m, 4H), 2.52–2.18 (m, 9H), 1.94–1.34 (m, 28H), 1.33–0.89 (m, 17H). ^13^C NMR (125 MHz, Benzene-*d_6_*) δ 293.7, 220.2, 146.0, 145.9, 145.7, 142.0, 141.9, 140.5, 140.4, 140.2, 140.0, 139.8, 139.6, 131.0, 129.8, 129.7, 129.6, 128.5, 128.3, 128.1, 127.5, 127.1, 127.0, 126.9, 116.7, 113.2, 60.4, 60.3, 60.2, 57.1, 56.6, 33.9, 33.7, 30.2, 30.1, 28.5, 27.6, 27.0, 22.6, 21.6, 21.5, 21.4, 21.3, 21.1, 15.5, 15,4. HRMS (ESI): [M^+^–Cl]_,_ found 1086.4988, requires C_62_H_84_N_3_O_3_PClRu 1086.4982. IR (KBr) 3053, 2922, 2848, 1724, 1608, 1589, 1539, 1480, 1444, 1418, 1377, 1356, 1266, 1252, 1173, 1094, 1058, 1028, 1006, 973, 847, 774, 752, 697.

### 3.6. Catalyst ***7***

To a solution of complex **6** (1.802 g, 1.67 mmol) in toluene (20 mL) was added two drops of concentrated hydrochloric acid and the mixture was stirred at rt for 20 min. The reaction mixture was then concentrated in vacuo. Purification by flash column chromatography (5–10% ethyl acetate/cyclohexane) gave a brown solid (1.260 g, 77%). ^1^H-NMR (500 MHz, Benzene-*d_6_*) δ 9.06 (ddd, *J* = 48.7, 30.7, 7.1 Hz, 1H), 8.00–7.59 (m, 3H), 7.38–7.18 (m, 3H), 7.14–6.80 (m, 6H), 6.42 (dd, *J* = 33.3, 27.5 Hz, 1H), 6.09–5.85 (m, 1H), 4.34–3.22 (m, 5H), 3.15–2.60 (m, 6H), 2.52–2.09 (m, 12H), 1.91–0.91 (m, 36H). ^13^C NMR (125 MHz, Benzene-*d_6_*) δ 293.9, 167.2, 145.6, 141.7, 141.5, 140.9, 139.8, 137.8, 137.2, 137.0, 136.6, 131.0, 130.9, 130.4, 129.5, 129.4, 129.3, 128.6, 128.3, 128.0, 126.8, 126.7, 126.6, 121.6, 118.8, 116.4, 83.8, 63.5, 56.0, 34.8, 34.5, 33.9, 30.3, 30.1, 29.9, 28.6, 28.5, 28.4, 27.6, 27.0, 22.7, 22.3, 21.6, 21.5, 21.4, 21.3, 20.8, 20.6, 20.2, 19.9, 19.7. HRMS (ESI): [M^+^–Cl]_,_ found 984.4324, requires C_57_H_74_N_3_OPClRu 984.4302. IR (KBr) 3436, 3053, 2923, 2848, 1945, 1738, 1608, 1590, 1538, 1485, 1444, 1419, 1378, 1357, 1266, 1249, 1203, 1174, 1112, 1040, 1029, 1005, 973, 847, 774, 752, 697.

### 3.7. 1,3-Bis(1-mesityl)-4,5-dihydro-4-[4-(dimethylamino)benzoyloxymethyl]-1H-imidazol-3-ium Tetrafluoroborate (***8***) 

To a solution of 1,3-bis(1-mesityl)-4,5-dihydro-4-hydroxymethyl-1*H*-imidazol-3-ium tetrafluoroborate (**4**) (1.650 g, 3.9 mmol) in pyridine (6 mL) was added 4-(dimethylamino)benzoyl chloride (1.00 g, 5.45 mmol). After stirring at rt for 24 h, the reaction mixture was concentrated in vacuo and dissolved in DCM (15 mL). The organic layer was washed with water (3 × 15 mL), dried over MgSO_4_ and concentrated. Crystallization from DCM/Et_2_O gave the product as a colorless solid (1.397 g, 63%). ^1^H-NMR (500 MHz, CDCl_3_) δ 8.30 (s, 1H), 7.75–7.68 (m, 2H), 7.00–6.93 (m, 4H), 6.64–6.57 (m, 2H), 5.37–5.27 (m, 1H), 4.76–4.64 (m, 2H), 4.34–4.22 (m, 2H), 3.07 (s, 6H), 2.42 (s, 3H), 2.38 (s, 3H), 2.37–2.30 (m, 3H), 2.28 (s, 3H), 2.28 (s, 3H), 1.57 (s, 3H). ^13^C NMR (125 MHz, CDCl_3_) δ 166.0, 159.8, 153.8, 140.9, 140.7, 135.6, 135.2, 131.6, 130.6, 130.4, 129.9, 128.5, 114.6, 110.6, 62.9, 60.7, 52.9, 40.0, 21.1, 21.0, 18.2, 18.0, 17.7. HRMS (ESI): M^+^_,_ found 484.2962, requires C_31_H_38_N_3_O_2_ 484.2964.

### 3.8. 1,3-Bis(1-mesityl)-4,5-dihydro-4-isonicotinoyloxymethyl-1H-imidazol-3-ium Tetrafluoroborate (***9***) 

To a solution of 1,3-bis(1-mesityl)-4,5-dihydro-4-hydroxymethyl-1*H*-imidazol-3-ium tetrafluoroborate (**4**) (1.70 g, 4.0 mmol) in pyridine (6.5 mL) was added isonicotinoyl chloride hydrochloride (0.997 g, 5.6 mmol). After stirring at rt for 16 h, the reaction mixture was concentrated in vacuo, dissolved in DCM (15 mL). The organic layer was washed with water (15 mL) and the aqueous layer was extracted with DCM (3 × 15 mL). The combined organic layers were dried over MgSO_4_ and concentrated. Crystallization from DCM/Et_2_O gave the product as a colorless solid (1.28 g, 60%). ^1^H-NMR (500 MHz, CDCl_3_) δ 8.73–8.66 (m, 2H), 8.22 (s, 1H), 7.52–7.47 (m, 2H), 6.92 (s, 3H), 6.85 (s, 1H), 5.48–5.38 (m, 1H), 4.78 (t, *J* = 12.6 Hz, 1H), 4.71 (dd, *J* = 12.7, 6.0 Hz, 1H), 4.45 (dd, *J* = 12.7, 3.0 Hz, 1H), 4.16 (dd, *J* = 12.8, 9.2 Hz, 1H), 2.36 (s, 3 H), 2.29 (br s, 6H), 2.27 (s, 3H), 2.26 (s, 3H), 2.21 (s, 3H). ^13^C NMR (125 MHz, CDCl_3_) δ 164.3, 159.6, 150.6, 140.8, 140.6, 135.8, 135.3, 130.5, 130.2, 129.8, 129.0, 128.2, 125.3, 122.6, 63.3, 62.2, 53.1, 21.0, 20.9, 18.0, 17.9, 17.6. HRMS (ESI): M^+^_,_ found 442.2491, requires C_28_H_32_N_3_O_2_ 442.2495.

### 3.9. Catalyst ***10***

To a solution of ligand **8** (1.89 g, 3.3 mmol) in toluene (165 mL) was added a solution of KHMDS in THF (13.2 mL, 3.3 mmol). After stirring at rt for 10 min, **Hov I** (1.82 g, 3.0 mmol) was added and stirred for another 10 min at rt and 40 min at 65 °C. Then CuCl (0.30 g, 3.0 mmol) was added and stirring and heating were continued for another 20 min. The reaction mixture was concentrated in vacuo. Purification by flash column chromatography (10–20% ethyl acetate/cyclohexane) gave a green solid (1.45 g, 60%). ^1^H-NMR (500 MHz, CDCl_3_) δ 16.51 (s, 1H), 7.85–7.78 (m, 2H), 7.51–7.44 (m, 1H), 7.06 (s, 3H), 7.02 (s, 1H), 6.96–6.88 (m, 1H), 6.85 (t, *J* = 7.4 Hz, 1H), 6.79 (d, *J* = 8.3 Hz, 1H), 6.63–6.53 (m, 2H), 4.90 (hept, *J* = 6.2 Hz, 1H), 4.84–4.75 (m, 1H), 4.54 (dd, *J* = 11.8, 4.5 Hz, 1H), 4.38–4.25 (m, 2H), 4.08 (t, *J* = 9.7 Hz, 1H), 3.02 (s, 6H), 2.77–2.21 (m, 18H), 1.28 (d, *J* = 6.1 Hz, 6H). ^13^C NMR (125 MHz, CDCl_3_) δ 298.0, 214.7, 166.6, 153.5, 152.2, 145.3, 139.7, 138.9, 138.8, 133.9, 131.7, 131.5, 130.0, 129.7, 129.6, 129.3, 122.8, 122.2, 115.7, 112.9, 110.6, 110.5, 74.9, 63.2, 61.2, 53.6, 40.0, 21.2–21.0 (m). HRMS (ESI): M^+^–Cl_,_ found 768.2513, requires C_41_H_49_N_3_O_3_ClRu 768.2506. IR (KBr) 3503, 2964, 2917, 1951, 1703, 1606, 1527, 1476, 1452, 1420, 1373, 1316, 1272, 1182, 1112, 1034, 938, 840, 828, 768, 745, 698, 574 cm^−1^.

### 3.10. Catalyst ***11***

To a solution of ligand **9** (2.04 g, 3.85 mmol) in toluene (50 mL) was added a solution of KHMDS in THF (16.8 mL, 4.2 mmol). After stirring at rt for 10 min, **Hov I** (2.11 g, 3.5 mmol) was added and stirred 40 min at 60 °C. Then CuCl (0.35 g, 3.5 mmol) was added and stirring and heating was continued for another 20 min. The reaction mixture was concentrated in vacuo. Purification by flash column chromatography (20% ethyl acetate/cyclohexane) gave a brown solid (1.066 g, 40%). ^1^H-NMR (500 MHz, CDCl_3_) δ 16.47 (s, 1H), 8.74 (s, 2H), 7.74 (s, 2H), 7.54–7.45 (m, 1H), 7.10–6.76 (m, 7H), 4.97–4.79 (m, 2H), 4.60 (dd, *J* = 11.8, 5.0 Hz, 1H), 4.47 (dd, *J* = 11.9, 4.2 Hz, 1H), 4.40–4.28 (m, 1H), 4.02 (t, *J* = 10.6, 1H), 2.94–2.25 (m, 9H), 1.96–1.66 (m, 3H), 1.46–1.17 (m, 12H). ^13^C NMR (125 MHz, CDCl_3_) δ 298.2, 190.2, 165.0, 152.3, 150.6, 145.3, 139.1, 136.3, 135.7, 130.2, 129.9, 129.7, 129.5, 128.3, 122.9, 123.0, 122.3, 120.4, 114.0, 112.9, 75.1, 65.1, 61.8, 50.8, 42.0, 22.00, 21.2–21.0 (m). HRMS (ESI): M^+^–Cl_,_ found 726.2044, requires C_38_H_43_N_3_O_3_ClRu 726.2036. IR (KBr) 3452, 2974, 2918, 1948, 1732, 1682, 1682, 1625, 1597, 1589, 1575, 1478, 1453, 1418, 1382, 1325, 1272, 1156, 1113, 1061, 1035, 937, 851, 754, 704, 574 cm^−1^.

## 4. Conclusions

In summary, two new ruthenium catalysts, **10** and **11**, which bear neutral, basic tag-moieties attached to the N-heterocyclic carbene ligand, were successfully obtained in a straightforward manner using the benzoylation strategy. These new tagged complexes are very stable and are catalytically active in a model metathesis reaction, however their activity depends strongly on the nature of the tag used. Catalyst **11**, bearing a strongly coordinating pyridine tag, is visibly less active in the model RCM reaction than its dimethylaniline analogue (**10**) and the parent Hoveyda–Grubbs catalyst, although the use of a mineral acid significantly offsets this difference. Having established a convenient way to prepare Hoveyda–Grubbs catalysts bearing basic tags in the NHC ligand, in a future study, we plan to immobilize them on selected solid supports and study catalysis under heterogeneous conditions.

## Data Availability

Data supporting the reported results are available from the corresponding author.

## Supplementary Materials

The following information may be found in the supplementary materials: 1. Unsuccessful attempts to direct esterification of complex **7**; 2: Metathesis reactions; 3. NMR spectra.

## Sample Availability

Not available.
