# Supplementary material for: Ruthenium Olefin Metathesis Catalysts Featuring N-Heterocyclic Carbene Ligands Tagged with Isonicotinic and 4-(Dimethylamino)benzoic Acid Rests: Evaluation of a Modular Synthetic Strategy
Source: Molecules. 2021 Aug 28;26(17):5220. doi: 10.3390/molecules26175220 (PMC8433898; doi:10.3390/molecules26175220)
Supplement: Supplementary file 1 [file molecules-26-05220-s001.zip › molecules-1302300-SI.pdf]

SUPPORTING INFORMATION

Ruthenium Olefin Metathesis Catalysts Featuring N-Heterocyclic Carbene Ligands Tagged with Isonicotinic and 4-(Dimethylamino)benzoic Acid Rests: Evaluation of a Modular Synthetic Strategy

Stefan Czarnocki,[a,#] Louis Monsigny,[a,#] Michał Sienkiewicz,[a]
Anna Kajetanowicz,[a,*] and Karol Grela[a,*]

[a] Biological and Chemical Research Centre, Faculty of Chemistry, University of Warsaw, Żwirki i Wigury 101, 02-089 Warsaw, Poland

[#] These authors contributed equally

E-mails of corresponding authors:
prof.grela@gmail.com (K. Grela);
a.kajetanowicz@uw.edu.pl (A. Kajetanowicz)

Table of contents

1. Unsuccessful attempts to direct esterification of complex **7** 2
 1.1. With tricyclohexylphosphine 2
 1.2. With pyridine 2
 1.3. With triethylamine 3
 1.4. Using DCC coupling method (Steglich esterification) 4
2. Metathesis reactions 5
 2.1 Ring-closing metathesis 5
 2.2 Cross metathesis 5
3. NMR spectra 7
4. References 17

1. Unsuccessful attempts to direct esterification of complex 7

1.1. With tricyclohexylphosphine

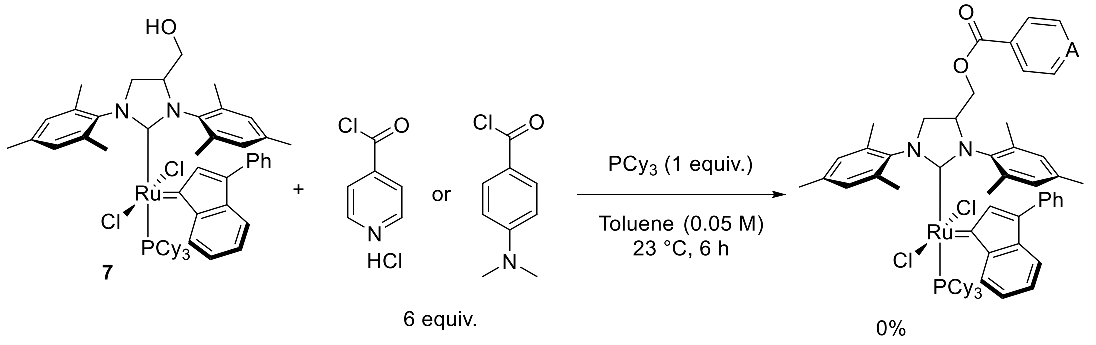

In a dry 10 mL round bottom flask complex **7** (50 mg, 0.051 mmol, 1 equiv.) was dissolved in 1 mL of dry degassed DCM (0.05 M). To this solution, tricyclohexylphosphine (14.2 mg, 0.05 mmol, 1 equiv.) was added, followed by isonicotinoyl chloride (54.4 mg, 0.306 mmol, 6 equiv.) or 4-dimethylaminobenzoyl chloride added in one portion (57.6 mg, 0.306 mmol, 6 equiv.). The progress of the reactions was monitored by TLC (20-50% AcOEt in hexane). In both cases after 6 h the starting material was completely decomposed and the desired products were not observed neither on TLC nor in ^{1}H NMR spectrum of the crude mixture.

1.2. With pyridine

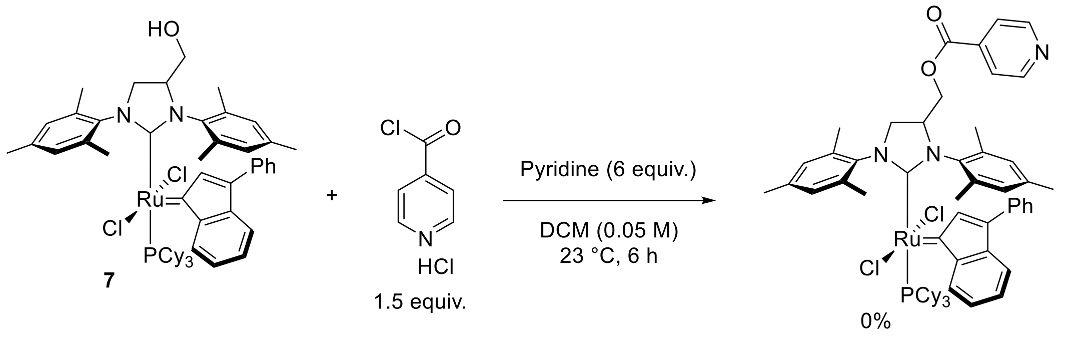

In a dry 10 mL round bottom flask complex **7** (50 mg, 0.051 mmol, 1 equiv.) was dissolved in 1 mL of dry degassed DCM (0.05 M). To this solution, pyridine (24.8 µL, 24.2 mg, 0.306 mmol, 6 equiv.) was added, followed by isonicotinoyl chloride added in one portion. (13.6 mg, 0.077 mmol, 1.5 equiv.). The progress of the reaction was monitored by TLC (50% AcOEt in hexane). After 6 h the starting material was completely decomposed and the desired product was not observed neither on TLC nor in ^{1}H NMR spectrum of the crude mixture.

1.3. With triethylamine

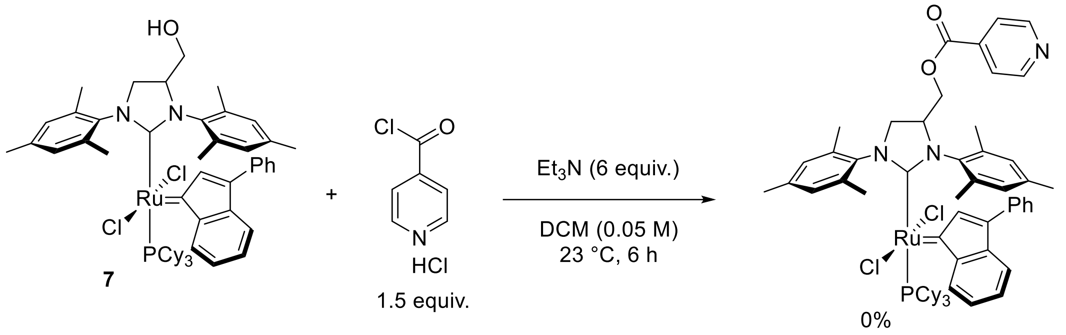

In a dry 10 mL round bottom flask complex **7** (50 mg, 0.051 mmol, 1 equiv.) was dissolved in 1 mL of dry degassed DCM (0.05 M). To this solution triethylamine (42.6 µL, 31 mg, 0.306 mmol, 6 equiv.) was added, followed by isonicotinoyl chloride added in one portion (13.6 mg, 0.077 mmol, 1.5 equiv.). The progress of the reaction was monitored by TLC (50% AcOEt in hexane). After 6 h of reaction, the starting material was consumed and the expected product was observed on TLC and in ^{1}H NMR spectrum. Unfortunately, the product was decomposed during purification on column chromatography (10% AcOEt in hexane to 20% AcOEt in hexane).

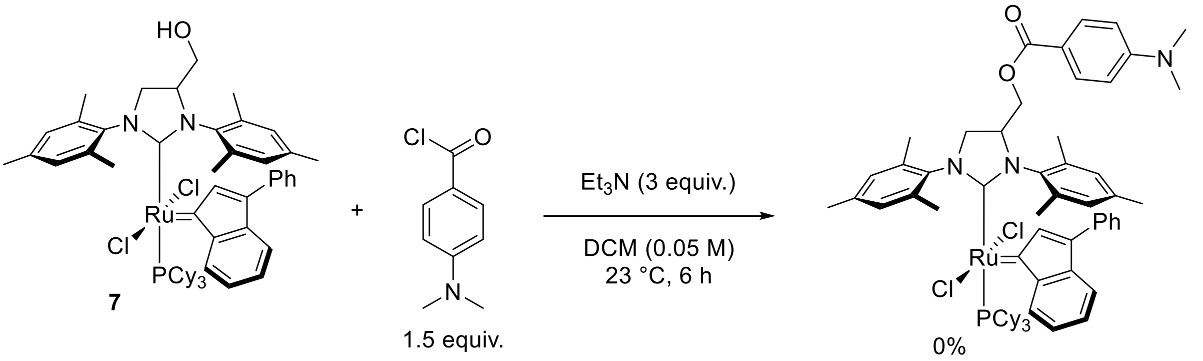

In a dry 10 mL round bottom flask complex **7** (50 mg, 0.051 mmol, 1 equiv.) was dissolved in 1 mL of dry degassed DCM (0.05 M). To this solution triethylamine (21.8 µL, 16 mg, 0.150 mmol, 3 equiv.) was added, followed by 4-dimethylaminobenzoyl chloride added in one portion (14.1 mg, 0.077 mmol, 1.5 equiv.). The progress of the reaction was monitored by TLC (20% AcOEt in hexane). After 6 h of reaction, the starting material was consumed and the expected product was observed on TLC and in ^{1}H NMR spectrum. Unfortunately, the product was decomposed during purification on column chromatography (10% AcOEt in hexane to 20% AcOEt in hexane).

1.4.Using DCC coupling method (Steglich esterification)

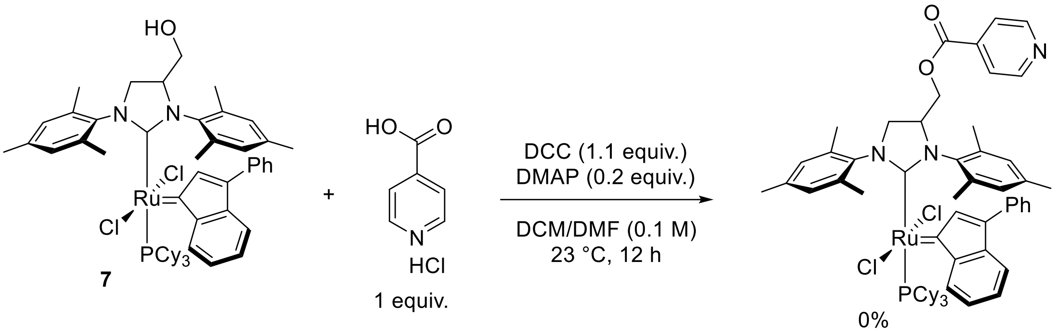

In a dry 25 mL round bottom flask under argon, the isonicotinic acid (12.6 mg, 0.102 mmol, 1 equiv.) was dissolved in 1 mL of dry degassed DCM/DMF mixture (v/v: 7/3) (0.1 M). To this solution, 4-*N,N*-dimethylaminopyridine DMAP (2.52 mg, 0.0204 mmol, 0.2 equiv.) was added, followed by the complex **7** added in one portion. (100 mg, 0.102 mmol, 1 equiv.). Dicyclohexylcarbodiimide (DCC, 23.9 mg, 0.112 mmol, 1.1 equiv.) was then added to the reaction mixture and the solution was stirred for 3 h. The progress of the reaction was monitored by TLC (20% AcOEt in hexane) and after 12 h of reaction, the starting material was decomposed and the expected product was not observed neither on TLC nor in ^{1}H NMR spectrum. Furthermore, no precipitation has been observed under these conditions highlighting the absence of the formation of urea and consequently, the absence of reaction.

2. Metathesis reactions

2.1 Ring-closing metathesis

(5-(5-((2,5-dihydro-1*H*-pyrrol-1-yl)sulfonyl)-2-ethoxyphenyl)-1-methyl-3-propyl-1,6-dihydro-7*H*-pyrazolo[4,3-*d*]pyrimidin-7-one), **P2**

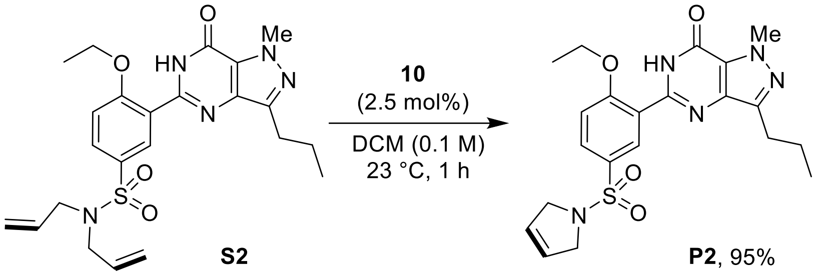

Sildenafil derivative **S2** (96 mg, 0.2 mmol, 1 equiv.) was dissolved in anhydrous DCM (1 mL) under argon atmosphere followed by addition of catalyst solution in anhydrous DCM (4.0 mg/mL, 1.0 mL, 4.0 mg, 0.005 mmol, 2.5 mol%). The resulting solution was stirred at 23 °C and monitored using TLC (stationary phase: SiO_2, eluent: EtOAc/*n*-hexane 50:50 v/v). After reaction completion, all volatiles were removed under reduced pressure and the crude product was purified using column chromatography (stationary phase: SiO_2, eluent: EtOAc/*n*-hexane 50:50 v/v). The product was obtained as a white solid (84 mg, 95%).

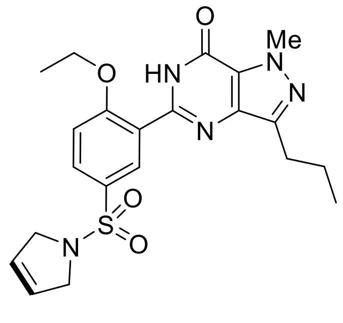

^{1}H NMR (400 MHz, $CDCl_3$) δ: 10.83 (bs, 1H), 8.88 (d, *J* = 2.5 Hz, 1H), 7.91 (dd, *J* = 8.8, 2.5 Hz, 1H), 7.14 (d, *J* = 8.7 Hz, 1H), 5.69 (s, 2H), 4.36 (q, *J* = 7.0 Hz, 2H), 4.26 (s, 3H), 4.18 (s, 4H), 2.93 (t, *J* = 7.6 Hz, 2H), 1.86 (sextet, *J* = 7.4 Hz, 2H), 1.63 (t, *J* = 7.0 Hz, 3H), 1.02 (t, *J* = 7.4 Hz, 3H).

^{13}C NMR (101 MHz, $CDCl_3$) δ: 159.3, 153.8, 147.1, 146.7, 138.5, 131.5, 131.1, 130.7, 125.6, 124.6, 121.3, 113.3, 66.2, 55.1, 38.4, 27.8, 22.4, 14.7, 14.2. Spectral data are in agreement with those reported in the literature [1].

2.2 Cross metathesis

Methyl 7-((tert-butyldimethylsilyl)oxy)hept-2-enoate, **P3**

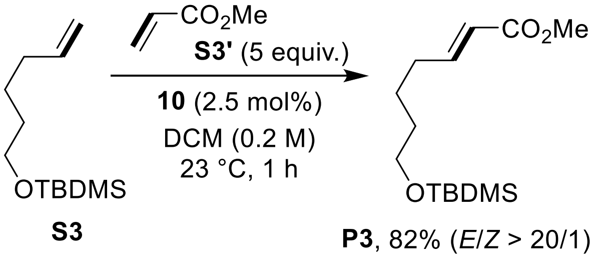

In a dry 10 mL round bottom flask filled with argon, *tert*-butyl(hex-5-en-1-yloxy)dimethylsilane (**S3**, 42.9 mg, 0.2 mmol, 1 equiv.) and the cross partner methyl acrylate (**S3'**, 91 µL,, 87 mg, 1 mmol, 5 equiv.) were dissolved in dry DCM (1 mL) under argon atmosphere. Catalyst solution

in anhydrous DCM (4.0 mg/mL, 1.0 mL, 4.0 mg, 0.005 mmol, 2.5 mol%) was added and the resulting solution was stirred at 23 °C. The progress of the reaction was monitored using TLC through the disappearance of the **S3** (eluent: hexane). After reaction completion, all the volatiles were removed under reduced pressure and the product was purified using column chromatography (stationary phase: SiO_2, eluent: 0 to 5% EtOAc/*n*-hexane). The product was obtained as a colorless oil (45 mg, 82%, *E*/*Z* > 99:1).

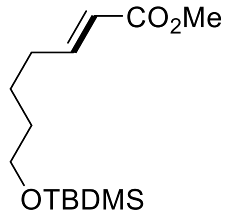

^{1}H NMR (400 MHz, $CDCl_3$) δ: 6.97 (dt, *J* = 15.7, 7.0 Hz, 1H), 5.82 (dt, *J* = 15.7, 1.6 Hz, 1H), 3.72 (s, 3H), 3.63 – 3.59 (m, 3H), 2.22 (m, 2H), 1.58 – 1.46 (m, 5H), 0.89 (s, 9H), 0.04 (s, 6H). **^{13}C NMR (101 MHz, $CDCl_3$) δ:** 167.3, 149.7, 121.2, 62.9, 51.5, 32.3, 32.1, 26.1, 24.5, 18.5, -5.0. Spectral data are in agreement with those reported in the literature [2].

(4-((6R,12a*R*)-6-(benzo[*d*][1,3]dioxol-5-yl)-1,4-dioxo-3,4,6,7,12,12a-hexahydropyrazino[1',2':1,6]pyrido[3,4-*b*]indol-2(1*H*)-yl)but-2-en-1-yl acetate), **P4**

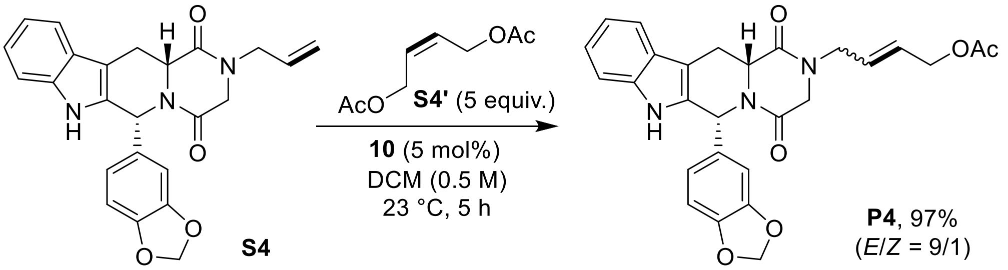

Tadalafil derivative **S4** (99.5 mg, 0.24 mmol, 1 equiv.) and the cross partner (*Z*)-1,4-acetoxybutene (**S4'**, 207 mg, 1.2 mmol, 5 equiv.) were dissolved in dry DCM (5 mL) under argon atmosphere. To the resulting mixture, the catalyst **10** (9.63 mg, 0.012 mmol, 5.0 mol%) was added in one portion and the mixture was stirred at 23 °C for 3 h. After reaction completion, the volatiles were removed under reduced pressure and the crude product was purified using column chromatography (stationary phase: SiO_2, eluent: 20 to 80% EtOAc/*n*-hexane) to furnish product **P4** as a white solid. The product was obtained as a white solid (114 mg, 97%, *E*/*Z* = 9:1).

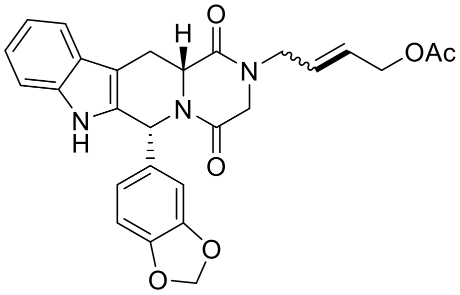

^{1}H NMR (101 MHz, $CDCl_3$) δ: 7.99 (s, 1H), 7.60 (dd, *J* = 7.1, 2.1 Hz, 1H), 7.28 (d, *J* = 2.0 Hz, 1H), 7.17 (dtd, *J* = 12.5, 7.0, 1.5 Hz, 2H), 6.83 (dd, *J* = 7.9, 1.9 Hz, 1H), 6.72 (d, *J* = 1.9 Hz, 1H), 6.68 (d, *J* = 7.9 Hz, 1H), 6.16 (s, 1H), 5.93 – 5.83 (m, 2H), 5.82 – 5.65 (m, 2H), 4.56 (d, J = 5.1 Hz, 2H), 4.32 (ddd, *J* = 11.4, 4.7, 1.3 Hz, 2H), 4.18 (dd, *J* = 15.2, 5.6 Hz, 1H), 4.06 – 3.97 (m, 2H), 3.76 (dd, J = 16.0, 4.6 Hz, 1H), 3.76 (dd, *J* = 16.0, 4.6 Hz, 1H), 3.22 (ddd, *J* = 16.0, 11.4, 1.5 Hz, 1H), 2.07 (s, 3H). **^{13}C NMR (101 MHz, $CDCl_3$) δ:** 170.6, 167.0, 166.2, 147.9, 147.1, 136.5, 135.2, 132.7, 129.3, 127.0, 126.1, 122.5, 120.7, 120.1, 118.6, 111.2, 108.2, 107.4, 106.4, 101.2, 63.7, 56.6, 56.2, 49.7, 47.4, 23.7, 20.9. Spectral data are in agreement with those reported in the literature [3].

3. NMR spectra

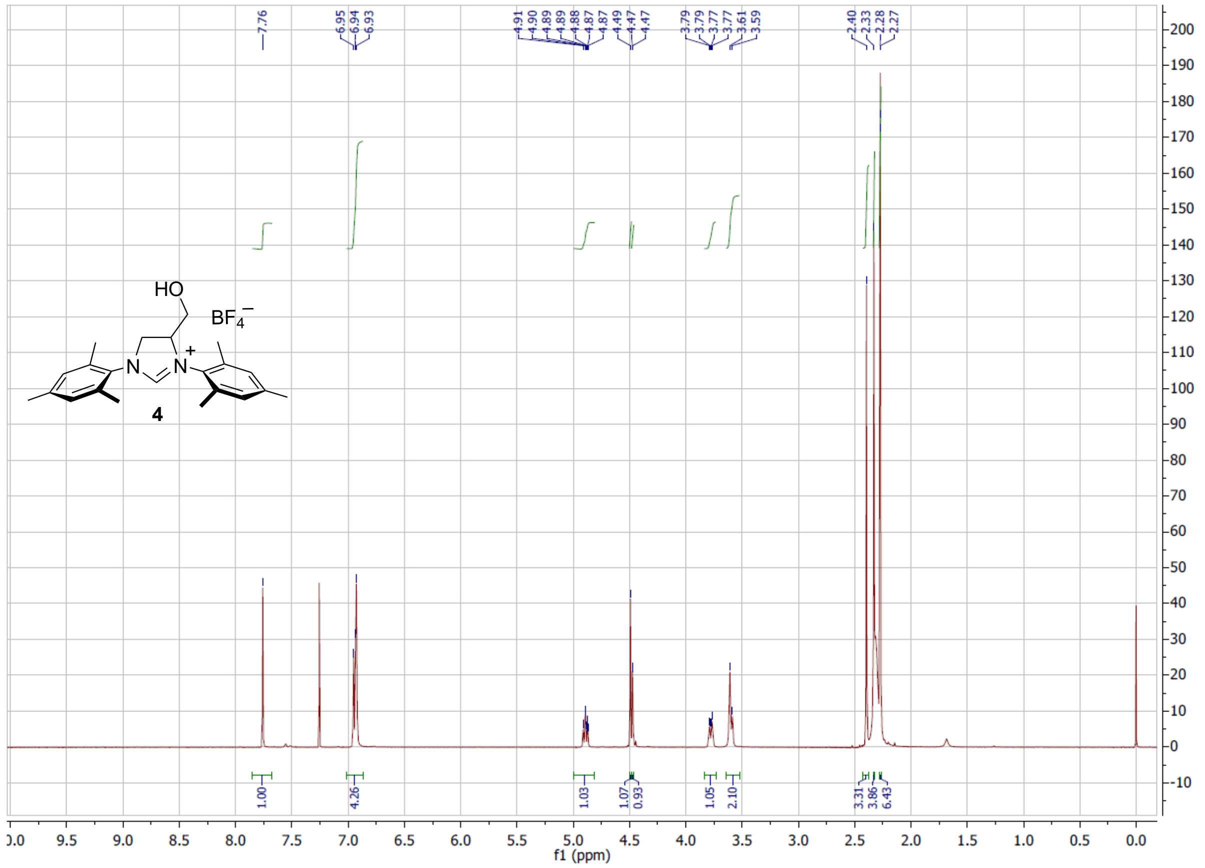

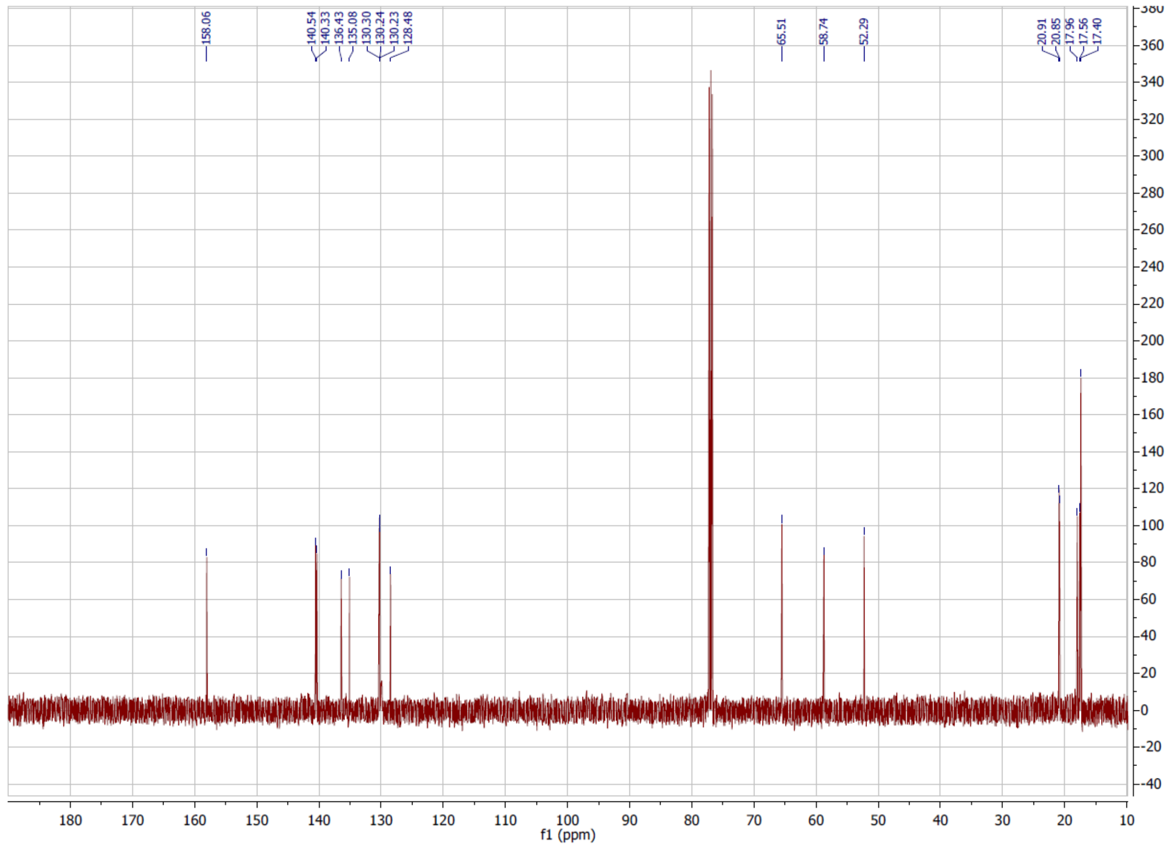

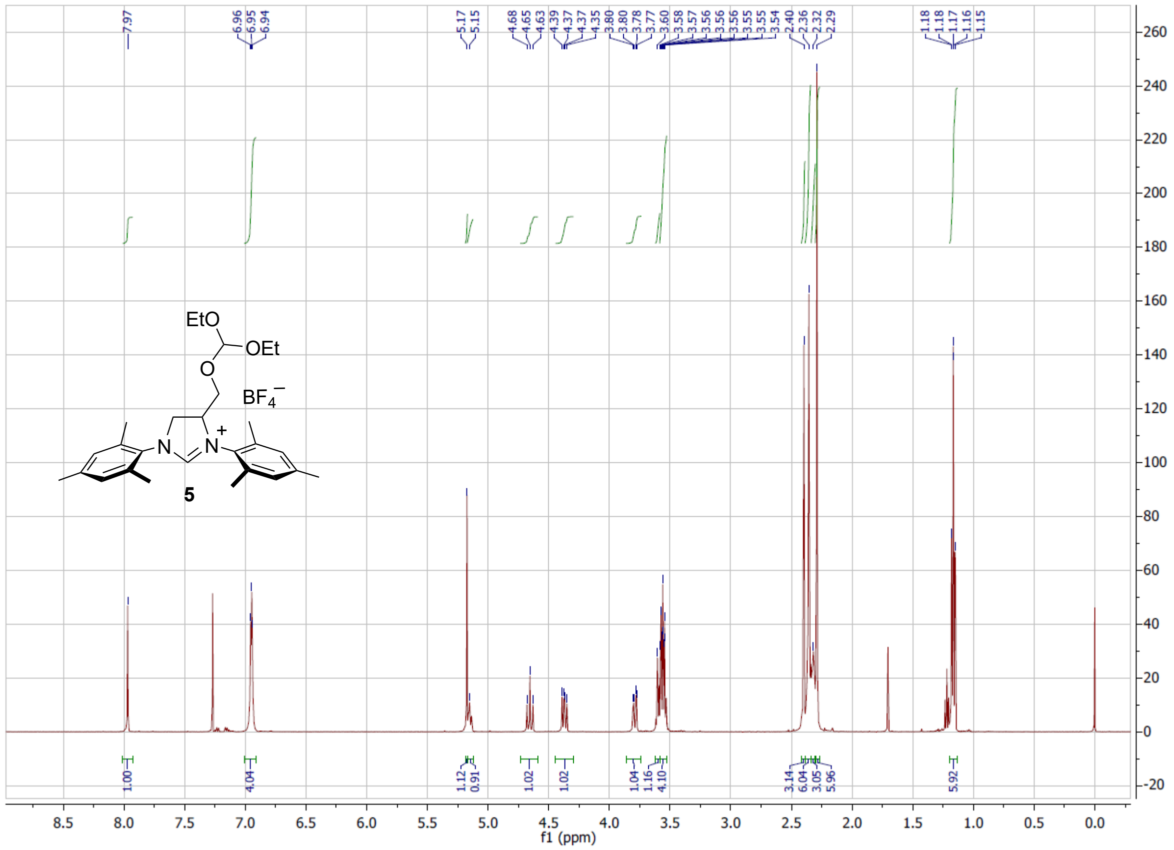

7.97
6.96
6.95
6.94
5.17
5.15
4.68
4.65
4.63
4.39
4.37
4.37
4.35
3.80
3.78
3.77
3.60
3.58
3.57
3.56
3.56
3.56
3.55
3.55
3.54
2.40
2.36
2.32
2.29
1.18
1.18
1.17
1.16
1.15
EtO
OEt
O
BF4−
N
N+
5
1.00
4.04
1.12
0.91
1.02
1.02
1.04
1.16
4.10
3.14
6.04
3.05
5.96
5.92
f1 (ppm)

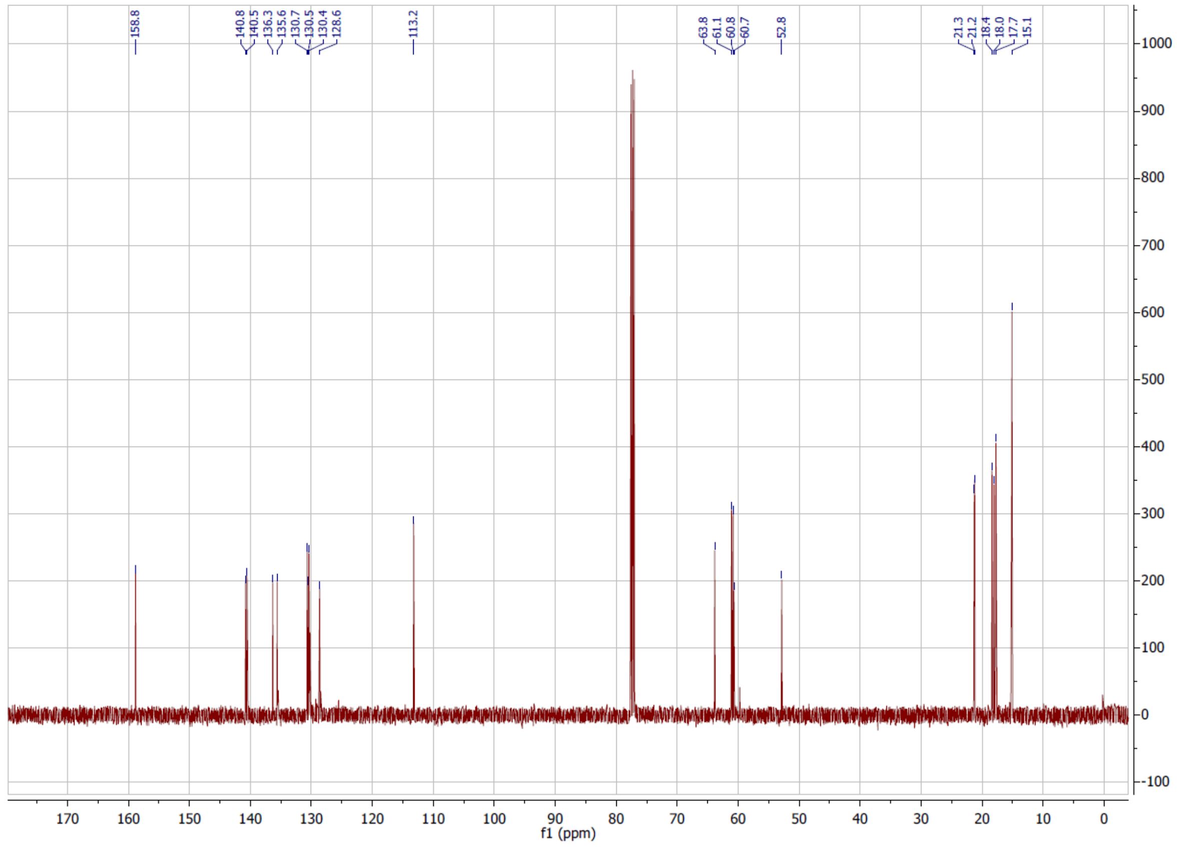

158.8
140.8
140.5
136.3
135.6
130.7
130.5
130.4
128.6
113.2
63.8
61.1
60.8
60.7
52.8
21.3
21.2
18.4
18.0
17.7
15.1
f1 (ppm)

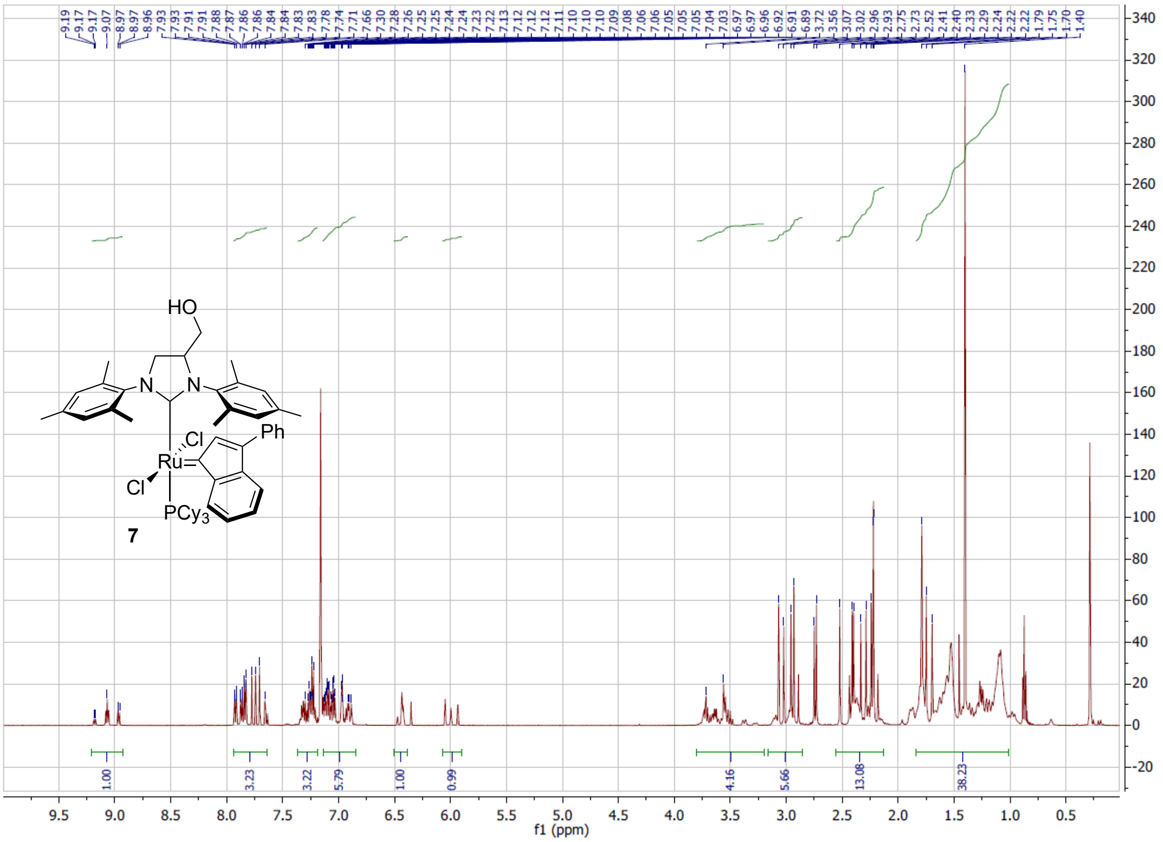
9.19 9.17 9.17 9.07 8.97 8.97 8.96 7.93 7.91 7.91 7.88 7.87 7.86 7.86 7.84 7.84 7.83 7.78 7.74 7.71 7.66 7.30 7.28 7.26 7.25 7.25 7.24 7.24 7.23 7.22 7.13 7.12 7.12 7.11 7.10 7.10 7.09 7.08 7.06 7.06 7.05 7.05 7.05 7.04 7.03 6.97 6.97 6.96 6.92 6.91 6.89 3.72 3.56 3.07 3.02 2.96 2.93 2.75 2.73 2.52 2.41 2.40 2.33 2.29 2.24 2.22 2.22 1.79 1.75 1.70 1.40
HO
N
N
Ph
Cl
Ru
Cl
PCy3
7
1.00
3.23
3.22
5.79
1.00
0.99
4.16
5.66
13.08
38.23
f1 (ppm)

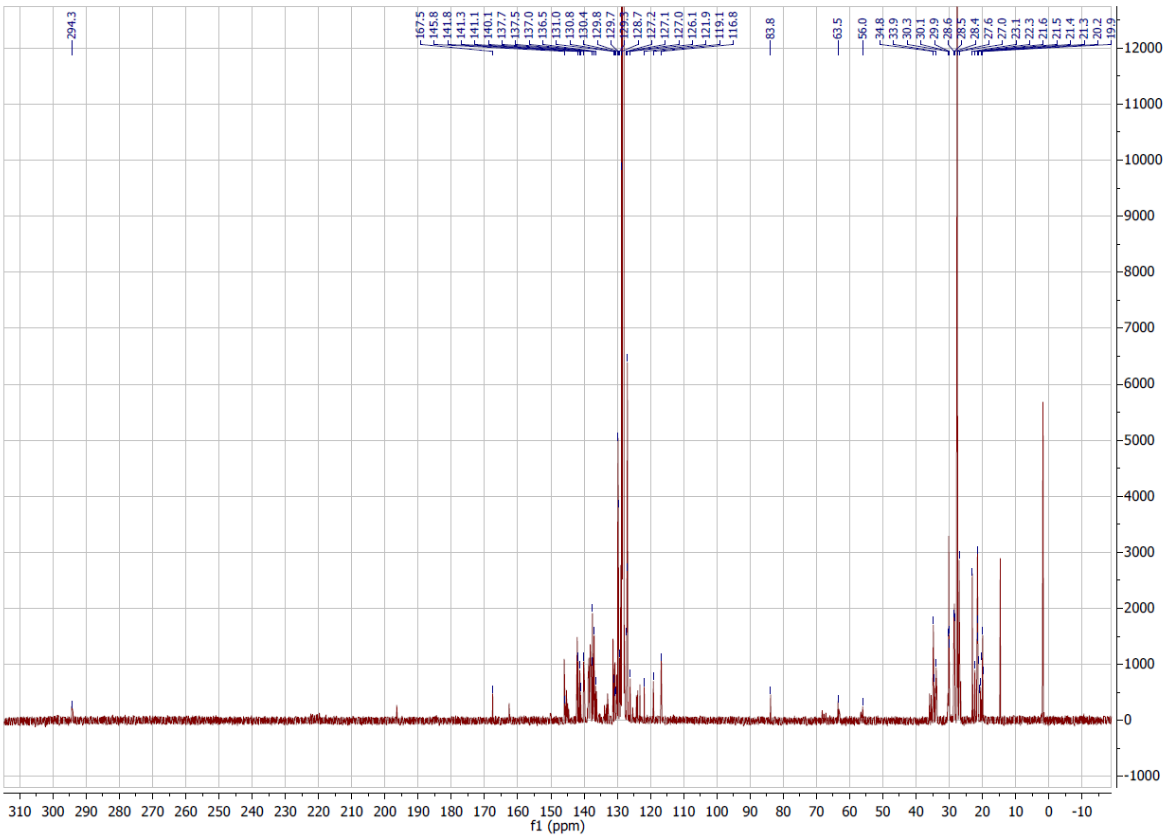
294.3 167.5 145.8 141.8 141.3 141.1 140.1 137.7 137.5 137.0 136.5 131.0 130.8 130.4 129.8 129.7 129.3 128.7 127.2 127.1 127.0 126.1 121.9 119.1 116.8 83.8 63.5 56.0 34.8 33.9 30.3 30.1 29.9 28.6 28.5 28.4 27.6 27.0 23.1 22.3 21.6 21.5 21.4 21.3 20.2 19.9
f1 (ppm)

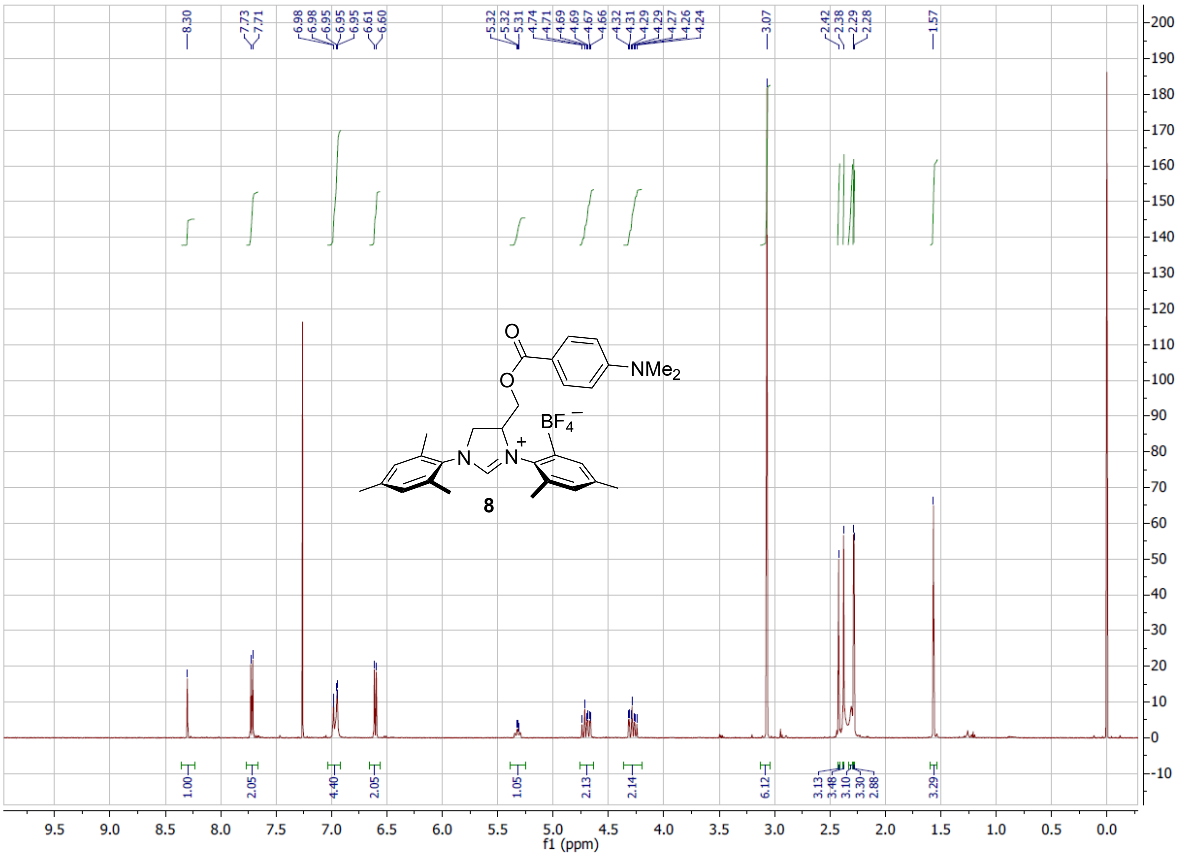
8.30
7.73 7.71
6.98 6.98 6.95 6.95 6.95 6.61 6.60
5.32 5.32 5.31 4.74 4.71 4.69 4.69 4.67 4.66 4.32 4.31 4.29 4.29 4.27 4.26 4.24
3.07
2.42 2.38 2.29 2.28
1.57
1.00 2.05 4.40 2.05 1.05 2.13 2.14 6.12 3.13 3.48 3.10 3.30 2.88 3.29
O
O
NMe2
BF4−
N
N+
8
f1 (ppm)

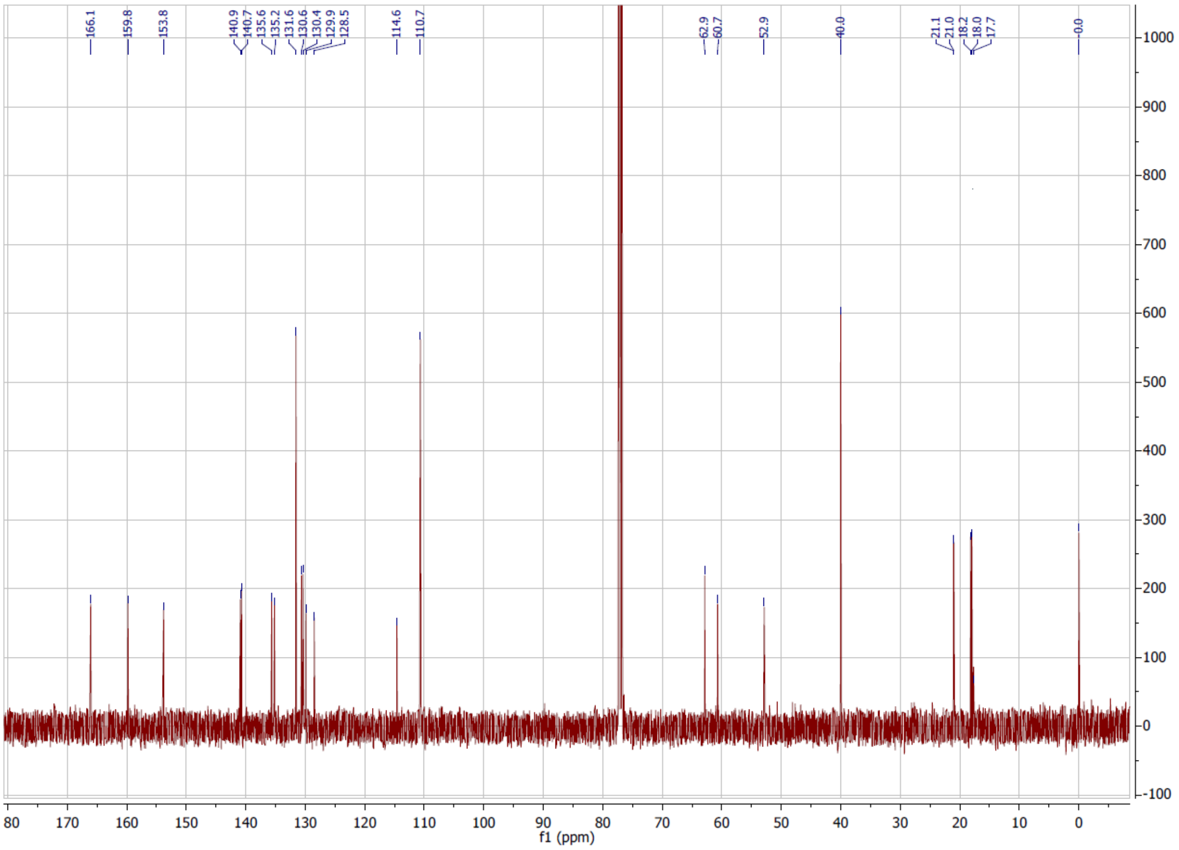
166.1 159.8 153.8
140.9 140.7 135.6 135.2 131.6 130.6 130.4 129.9 128.5
114.6 110.7
62.9 60.7 52.9 40.0
21.1 21.0 18.2 18.0 17.7
0.0
f1 (ppm)

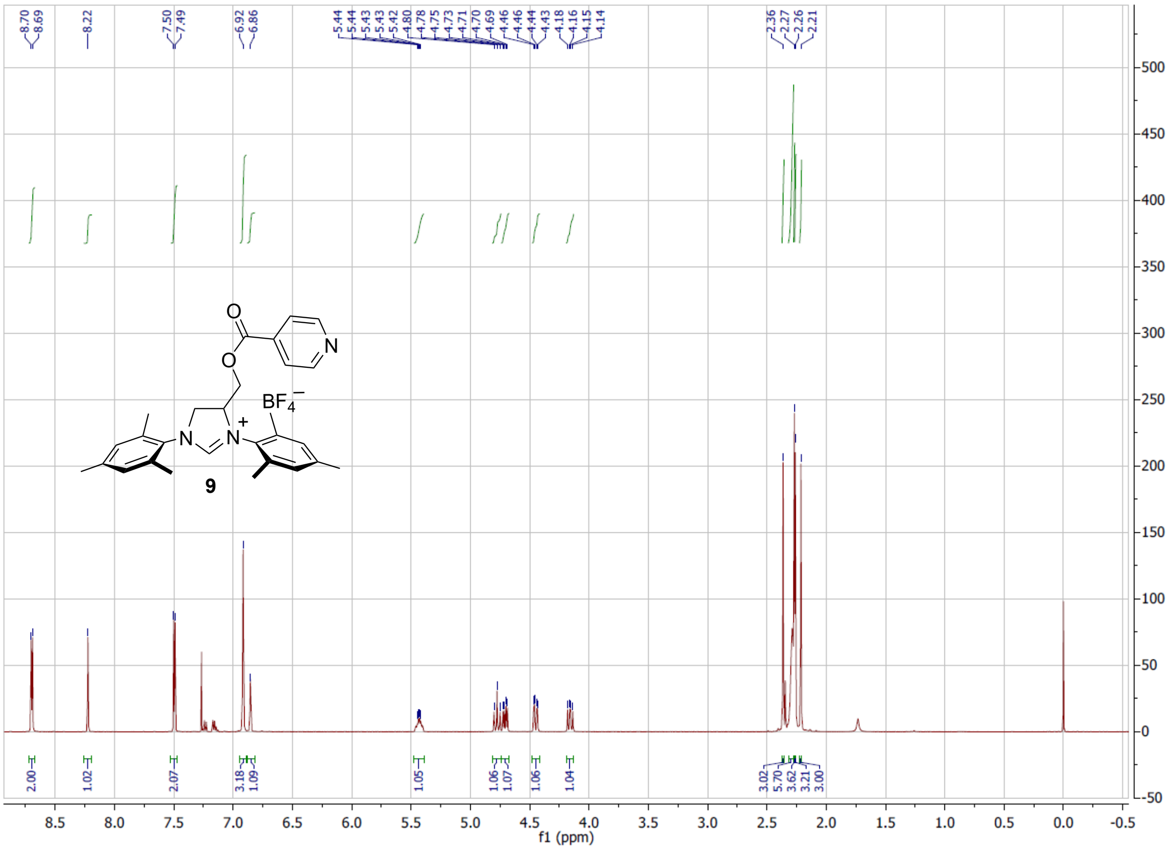
8.70
8.69
8.22
7.50
7.49
6.92
6.86
5.44
5.44
5.43
5.43
5.42
4.80
4.78
4.75
4.73
4.71
4.70
4.69
4.46
4.46
4.44
4.43
4.18
4.16
4.15
4.14
2.36
2.27
2.26
2.21
9
2.00
1.02
2.07
3.18
1.09
1.05
1.06
1.07
1.06
1.04
3.02
5.70
3.62
3.21
3.00
f1 (ppm)

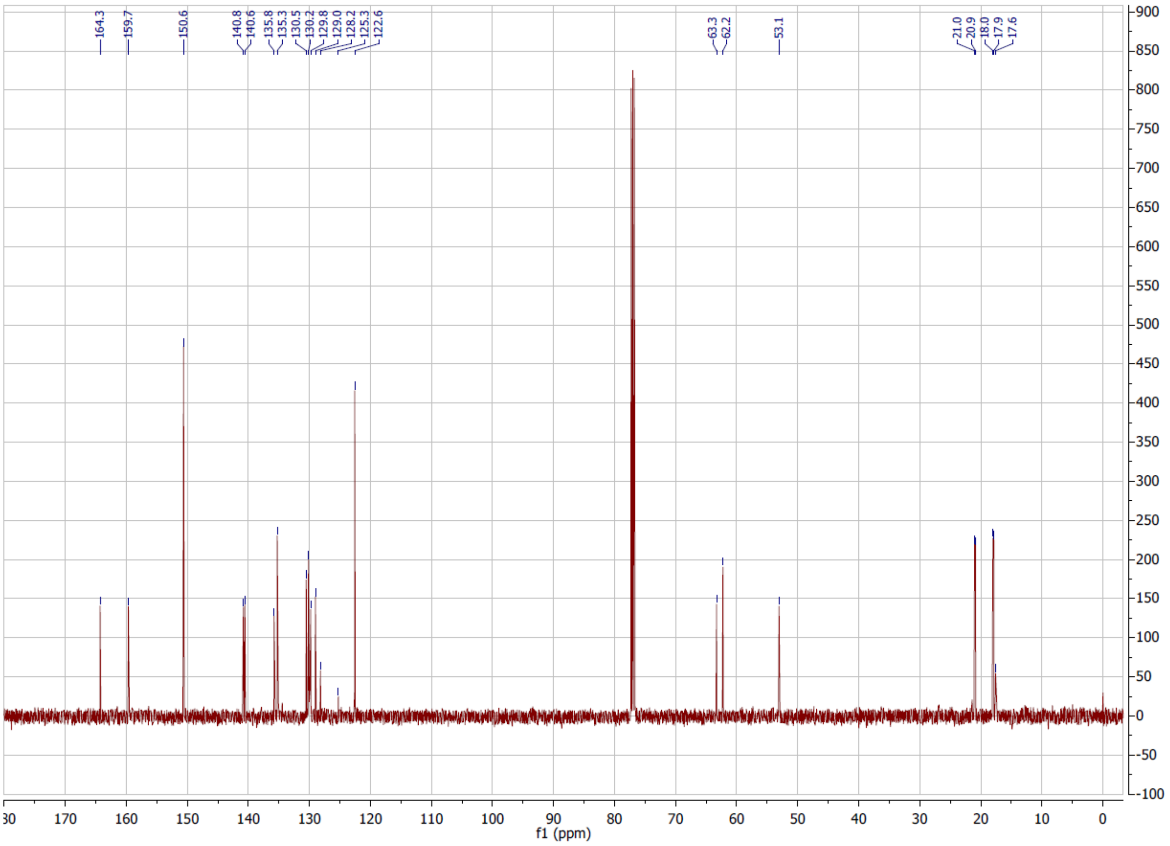
164.3
159.7
150.6
140.8
140.6
135.8
135.3
130.5
130.2
129.8
129.0
128.2
125.3
122.6
63.3
62.2
53.1
21.0
20.9
18.0
17.9
17.6
f1 (ppm)

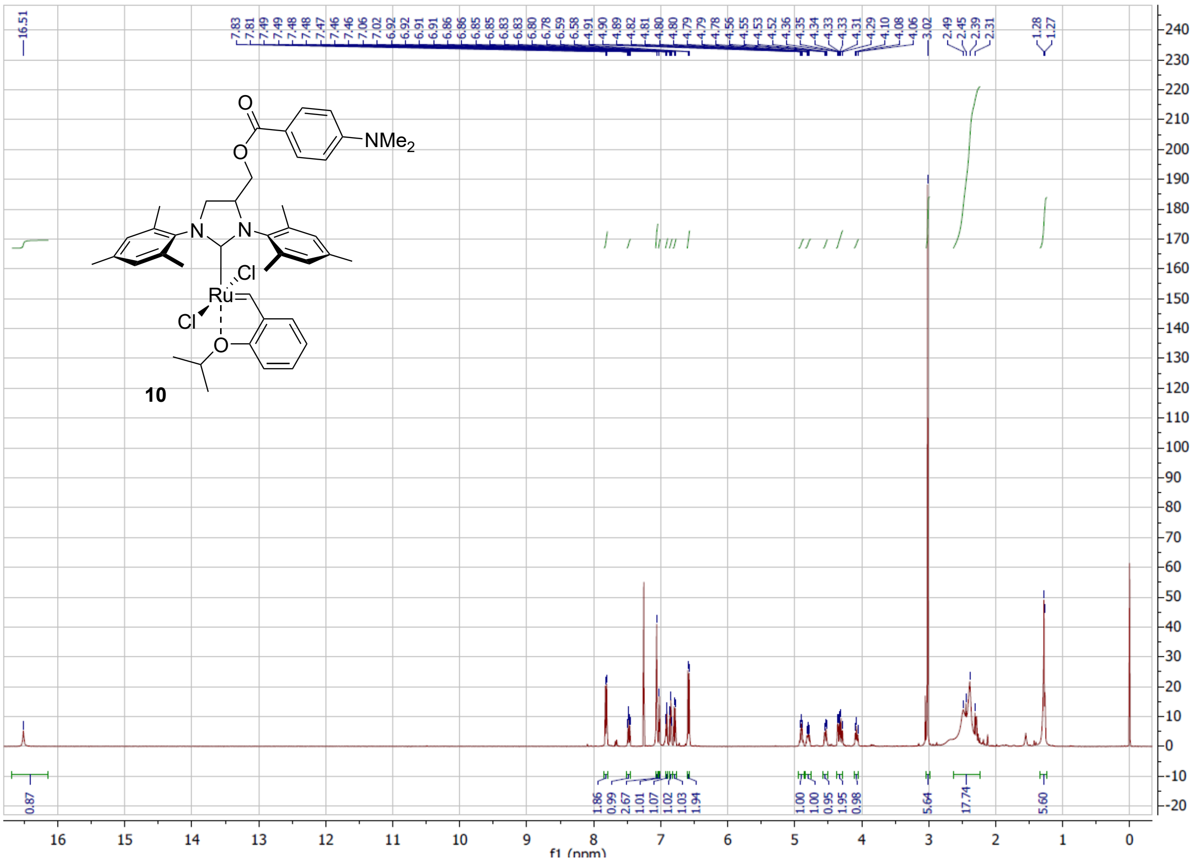

16.51
7.83 7.81 7.49 7.48 7.48 7.47 7.46 7.46 7.06 7.02 6.92 6.92 6.91 6.91 6.86 6.86 6.85 6.85 6.83 6.83 6.80 6.78 6.59 6.58 4.91 4.90 4.89 4.82 4.81 4.80 4.80 4.79 4.78 4.56 4.55 4.53 4.52 4.36 4.35 4.34 4.33 4.33 4.31 4.29 4.10 4.08 4.06 3.02 2.49 2.45 2.39 2.31 1.28 1.27
O
O
NMe2
N
N
Cl
Ru
Cl
O
10
0.87
1.86 0.99 2.67 1.01 1.07 1.02 1.03 1.94
1.00 1.00 0.95 1.95 0.98
5.64
17.74
5.60
f1 (ppm)

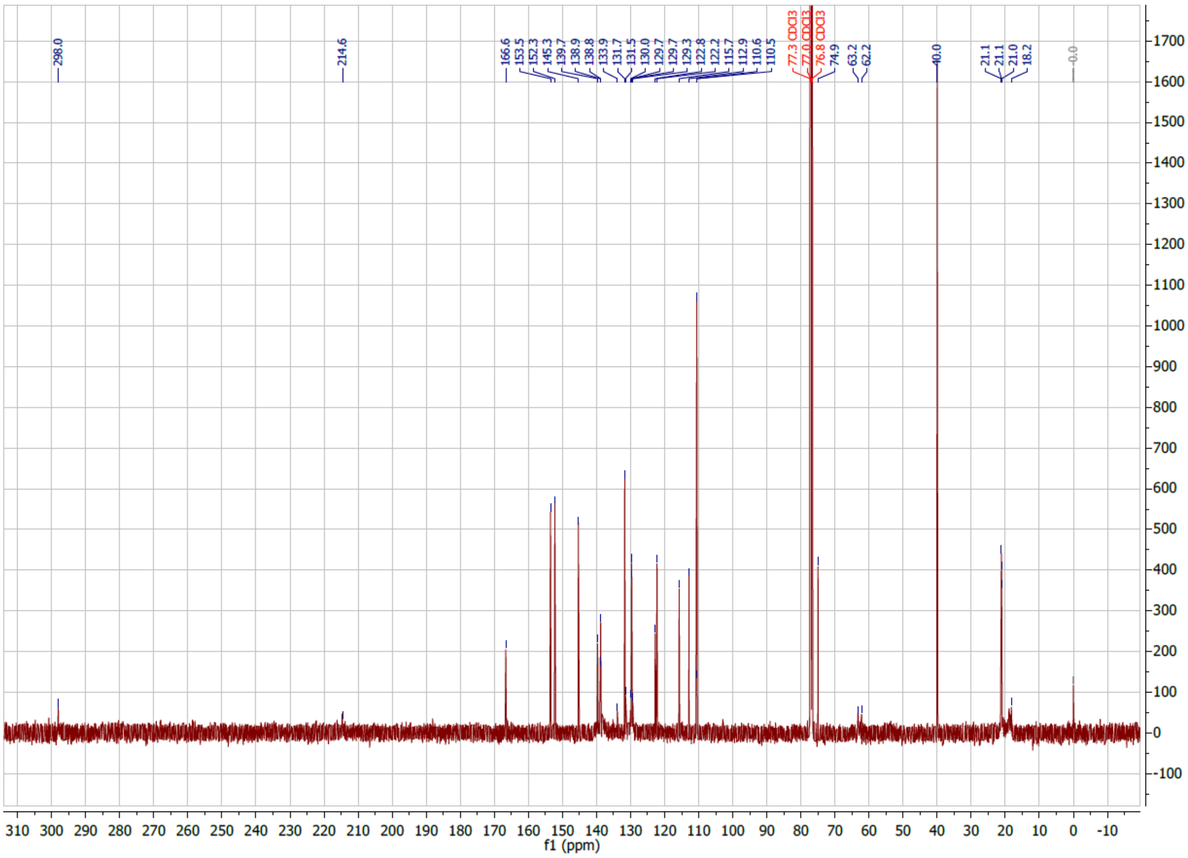

298.0
214.6
166.6 153.5 152.3 145.3 139.7 138.9 138.8 133.9 131.7 131.5 130.0 129.7 129.7 129.3 122.8 122.2 115.7 112.9 110.6 110.5
77.3 CDCl3 77.0 CDCl3 76.8 CDCl3
74.9
63.2 62.2
40.0
21.1 21.1 21.0 18.2
0.0
f1 (ppm)

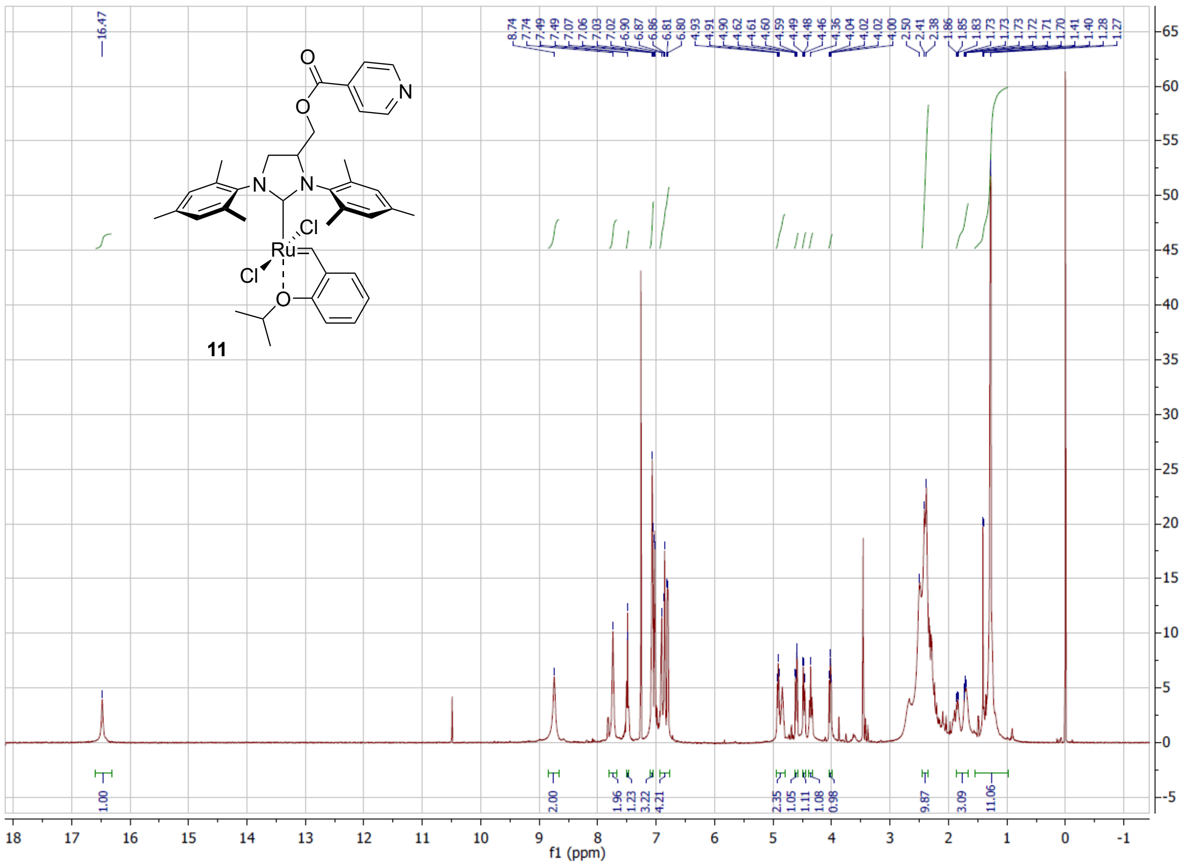

11

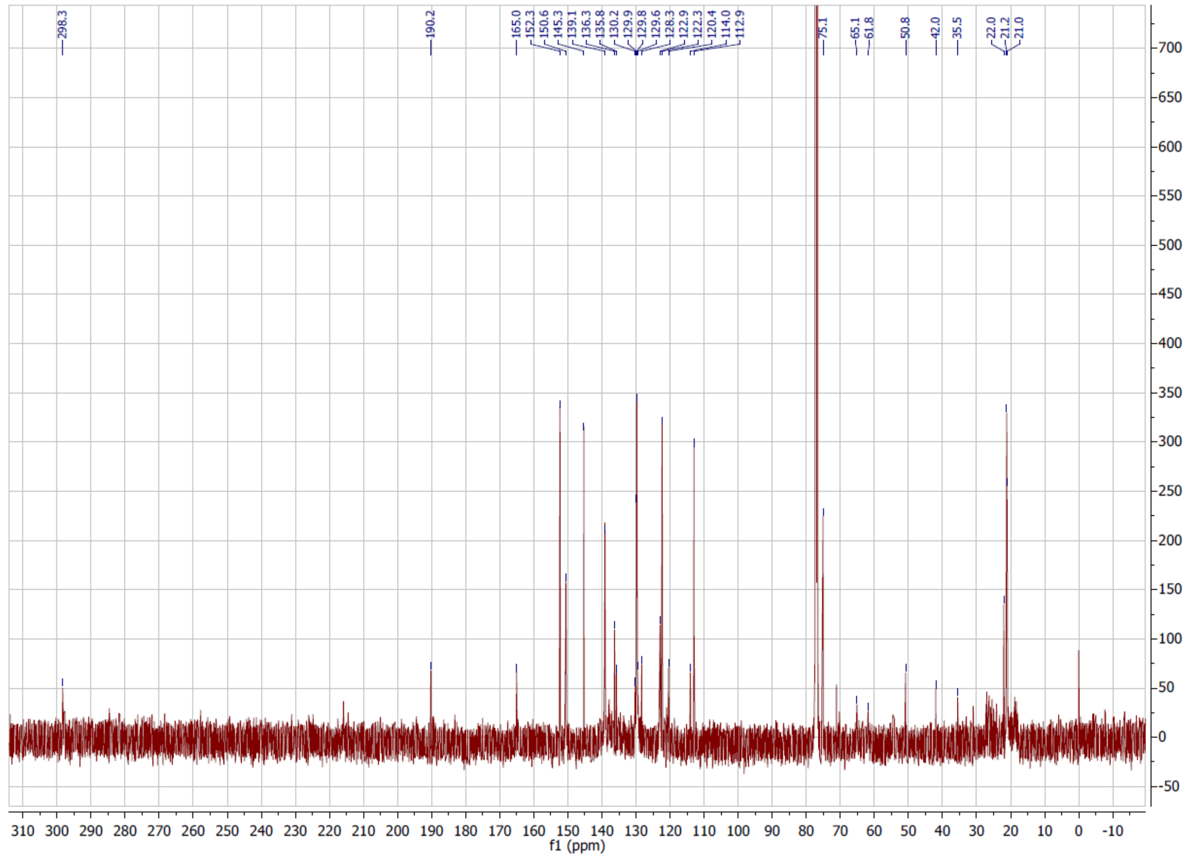

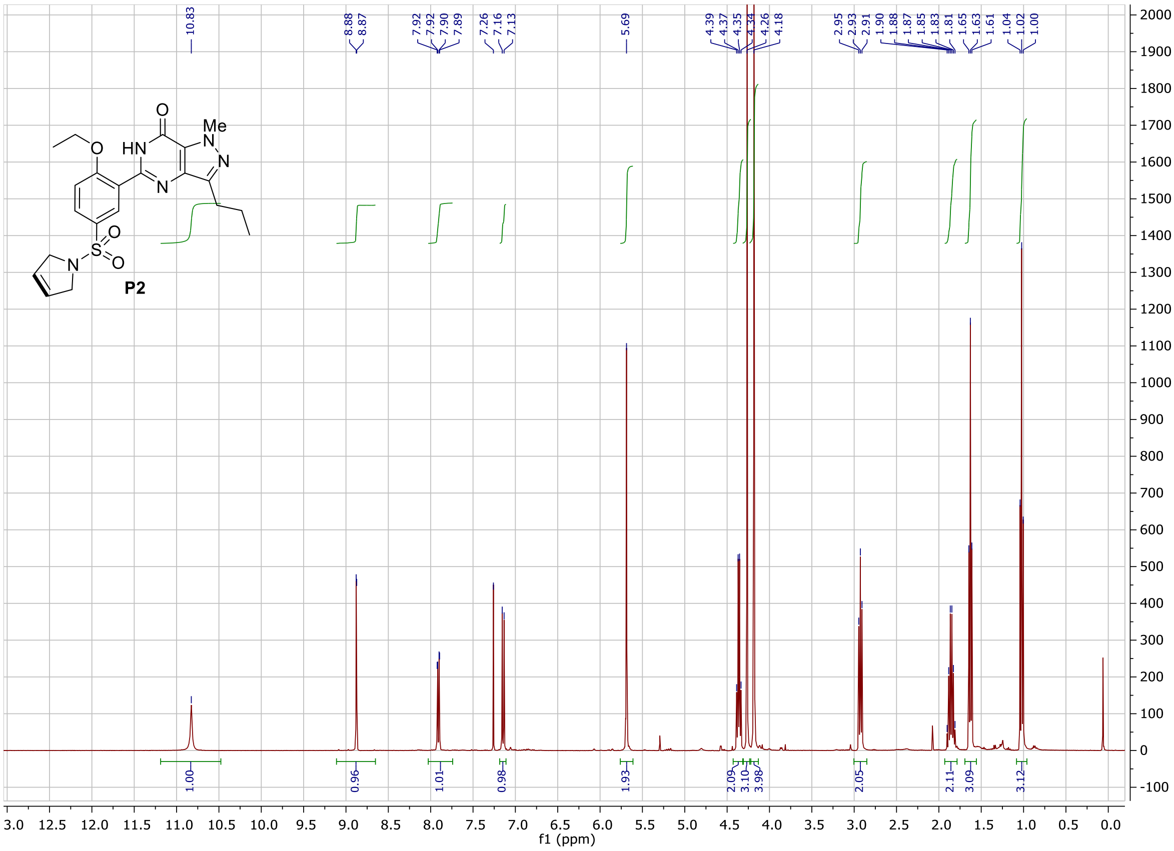

10.83
8.88
8.87
7.92
7.92
7.90
7.89
7.26
7.16
7.13
5.69
4.39
4.37
4.35
4.34
4.26
4.18
2.95
2.93
2.91
1.90
1.88
1.87
1.85
1.83
1.81
1.65
1.63
1.61
1.04
1.02
1.00
O
Me
HN
N
N
O
N
N
S
O
O
P2
1.00
0.96
1.01
0.98
1.93
2.09
3.10
3.98
2.05
2.11
3.09
3.12
f1 (ppm)

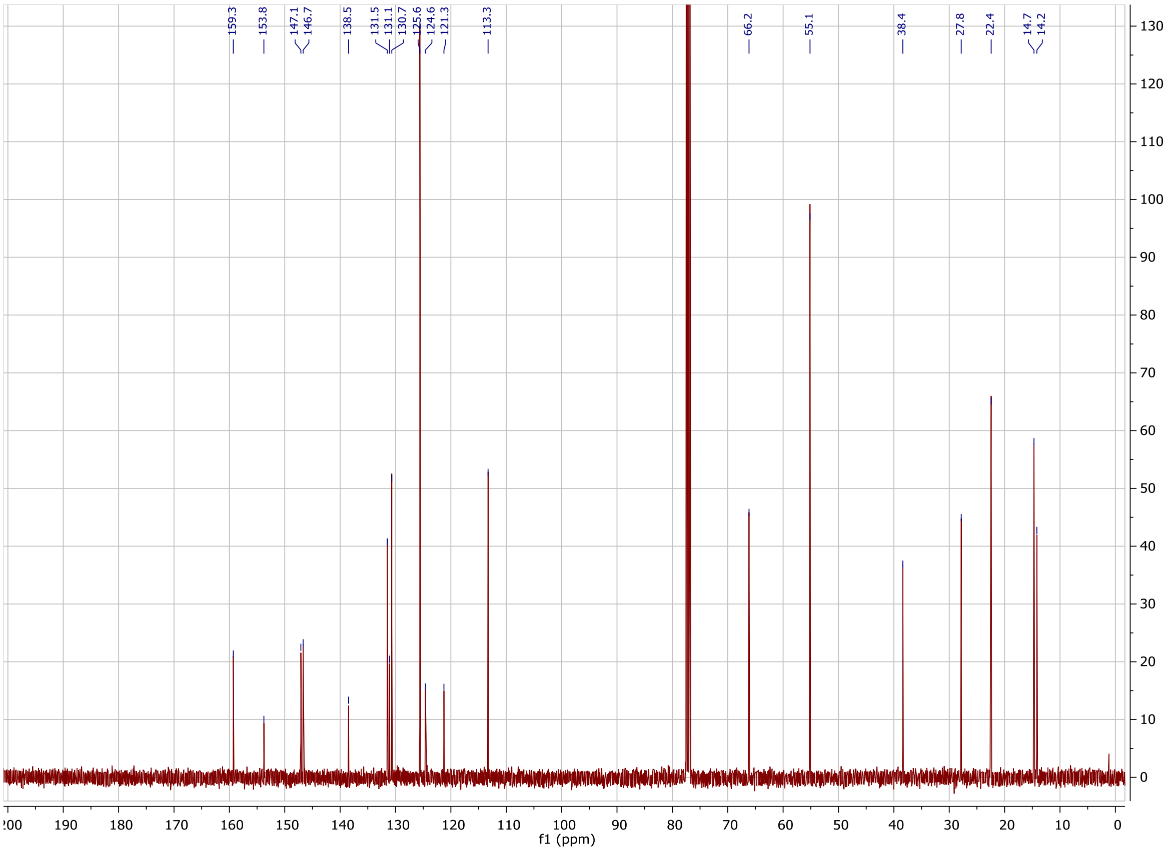

159.3
153.8
147.1
146.7
138.5
131.5
131.1
130.7
125.6
124.6
121.3
113.3
66.2
55.1
38.4
27.8
22.4
14.7
14.2
f1 (ppm)

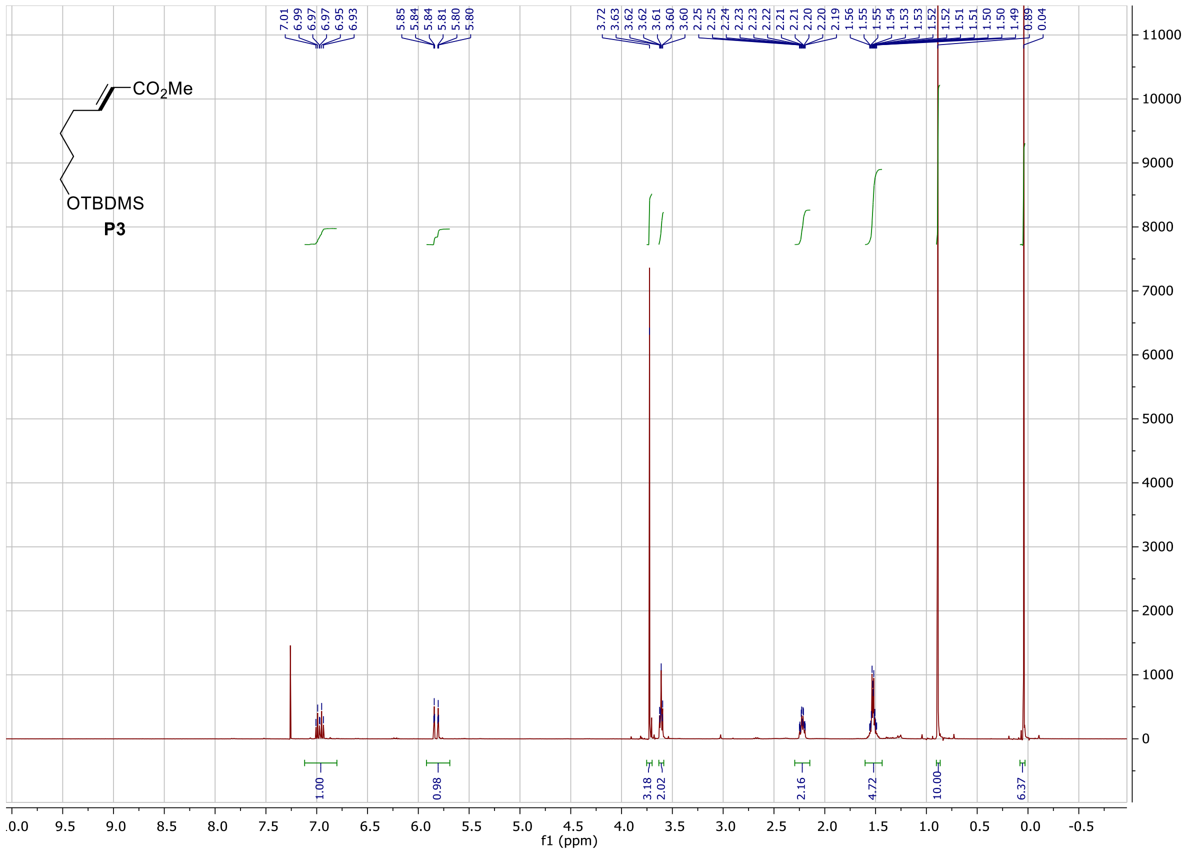
CO2Me
OTBDMS
P3
7.01 6.99 6.97 6.97 6.95 6.93
5.85 5.84 5.84 5.81 5.81 5.80
3.72 3.63 3.62 3.62 3.61 3.61 3.60 3.60
2.25 2.25 2.24 2.23 2.23 2.22 2.21 2.21 2.20 2.20 2.19
1.56 1.55 1.55 1.54 1.53 1.53 1.52 1.52 1.51 1.51 1.50 1.50 1.49
0.89 0.04
1.00
0.98
3.18
2.02
2.16
4.72
10.00
6.37
f1 (ppm)

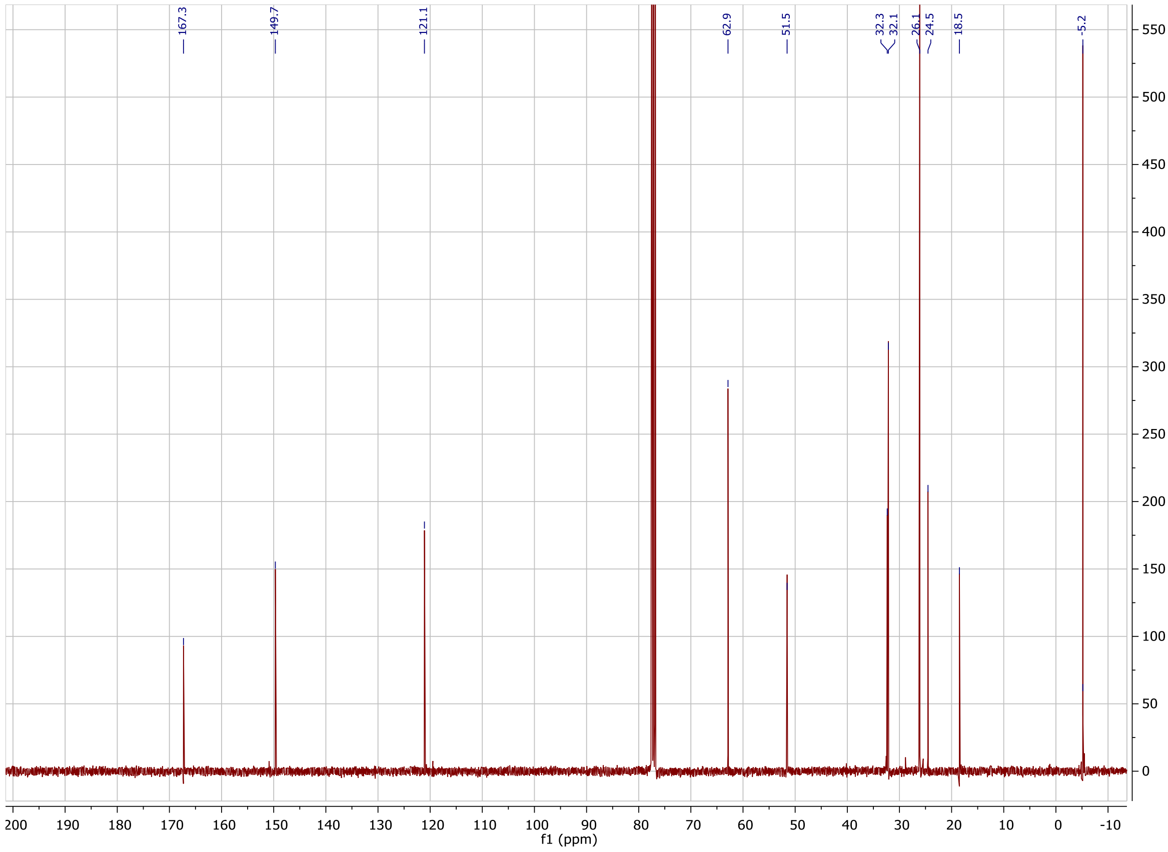
167.3
149.7
121.1
62.9
51.5
32.3
32.1
26.1
24.5
18.5
-5.2
f1 (ppm)

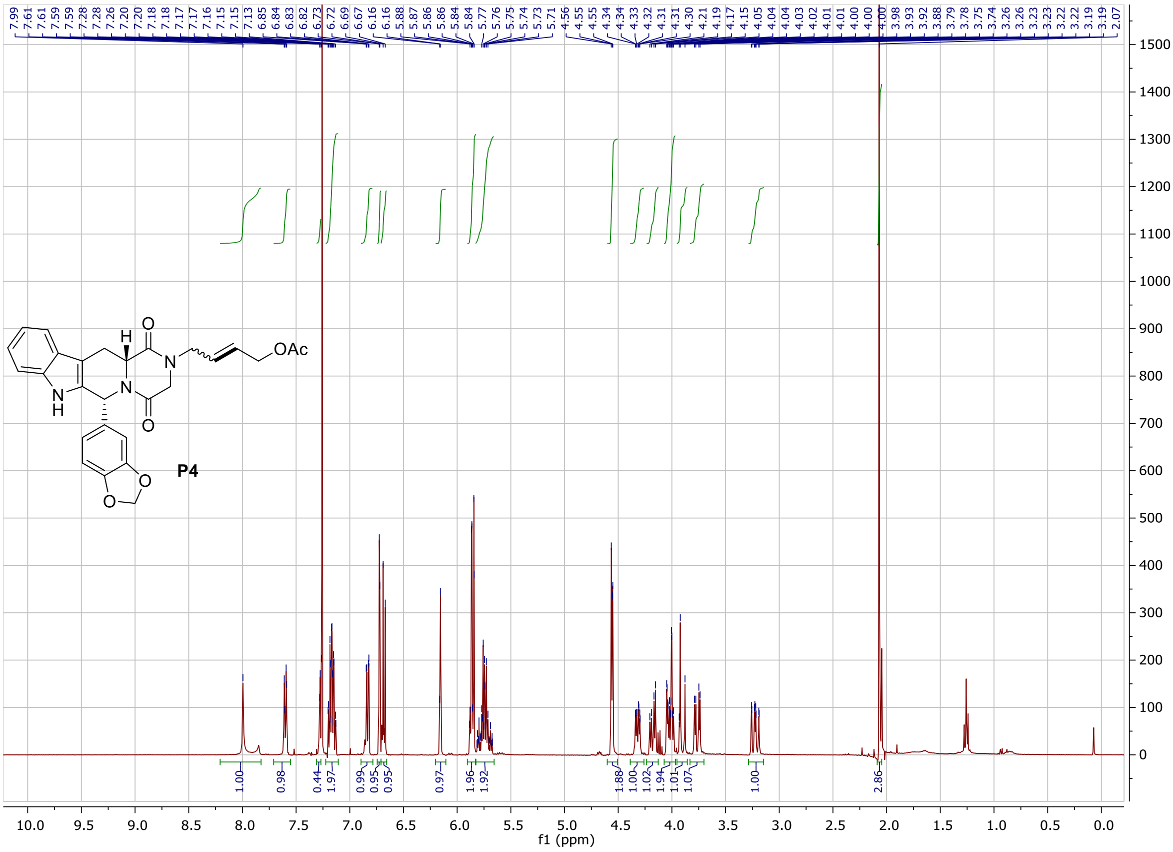

OAc
P4
f1 (ppm)

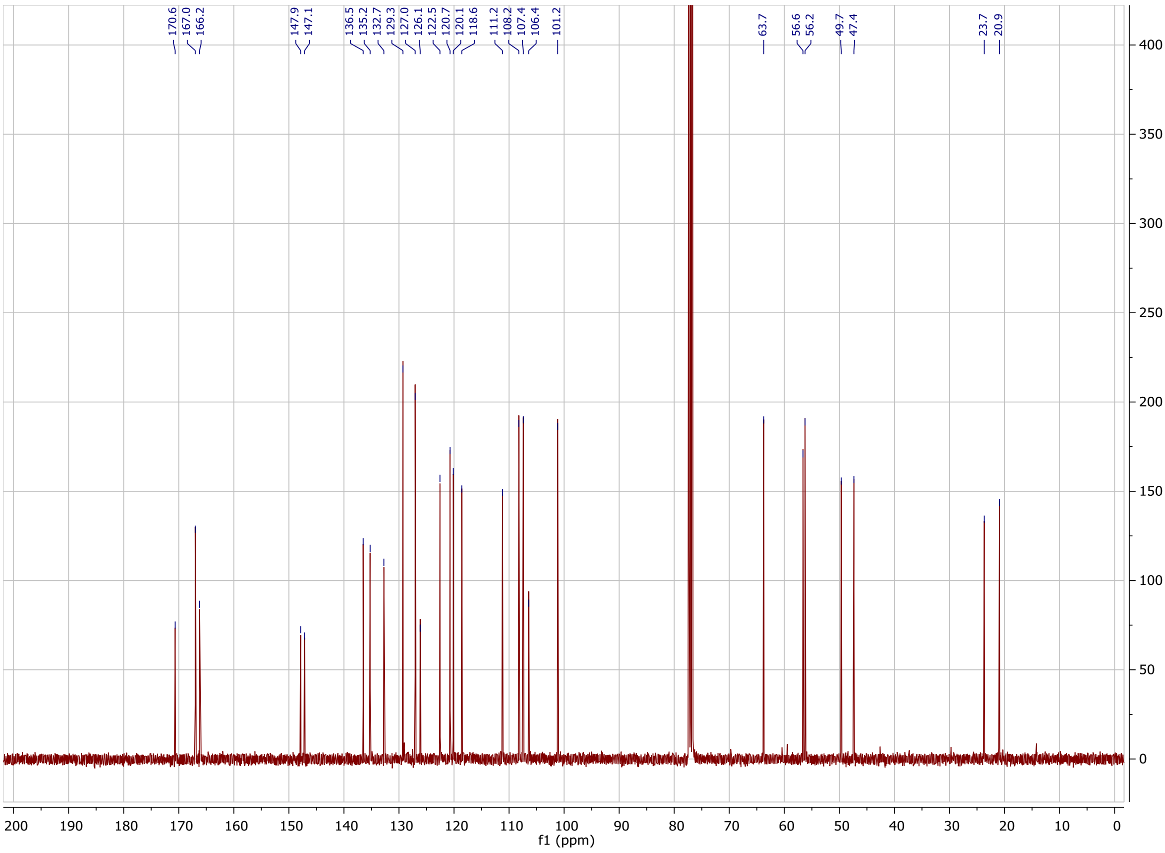

f1 (ppm)

4. References

1. Nienałtowski, T.; Krzesiński, P.; Baumert, M.E.; Skoczeń, A.; Suska-Kauf, E.; Pawłowska, J.; Kajetanowicz, A.; Grela, K. 4-Methyltetrahydropyran as a Convenient Alternative Solvent for Olefin Metathesis Reaction: Model Studies and Medicinal Chemistry Applications. *ACS Sustainable Chem. Eng.* **2020**, *8*, 18215-18223, doi:10.1021/acssuschemeng.0c06668.

2. Bieniek, M.; Bujok, R.; Cabaj, M.; Lugan, N.; Lavigne, G.; Arlt, D.; Grela, K. Advanced Fine-Tuning of Grubbs/Hoveyda Olefin Metathesis Catalysts: A Further Step toward an Optimum Balance between Antinomic Properties. *J. Am. Chem. Soc.* **2006**, *128*, 13652-13653, doi:10.1021/ja063186w.

3. Monsigny, L.; Piątkowski, J.; Trzybiński, D.; Wozniak, K.; Nienałtowski, T.; Kajetanowicz, A.; Grela, K. Activated Hoveyda-Grubbs Olefin Metathesis Catalysts Derived from a Large Scale Produced Pharmaceutical Intermediate—Sildenafil Aldehyde. *Adv. Synth. Catal.* **2021**, *in press*, doi:https://doi.org/10.1002/adsc.202100669.
